# Supplementary material for: Clinical outcomes of chikungunya: A systematic literature review and meta-analysis
Source: PLoS Negl Trop Dis. 2024 Jun 7;18(6):e0012254. doi: 10.1371/journal.pntd.0012254 (PMC11189168; doi:10.1371/journal.pntd.0012254)
Supplement: S1 Text — Containing the search strategy and PICOs of the studies included in the SLR. (DOCX) [file pntd.0012254.s001.docx]

**S1 Text. Literature search and study selection**

**Search string**

An Embase pilot search strategy for the clinical SLR is presented in Table 1. This Embase search strategy was adapted to search PubMed, see Table 2.

Table 1. Clinical SLR Embase search strategy (July 4, 2023)

| String Number | Query | Hits |
| --- | --- | --- |
| 1 | ‘chikungunya’/exp OR ‘chikungunya’:ab,ti | 8,282 |
| 2 | 'case study'/it OR 'case report'/it OR 'abstract report'/it OR editorial/it OR ‘veterinary clinical trial’/it OR letter/it OR note/it | 2,714,721 |
| 3 | #1 NOT #2 | 7,547 |

Table 2. Clinical SLR PubMed search strategy (July 4, 2023)

| String Number | Query | Hits |
| --- | --- | --- |
| 1 | “Chikungunya fever”[Mesh] OR “chikungunya”[tiab] | 6,056 |
| 2 | “case reports”[pt] OR editorial[pt] OR letter[pt] OR comment[pt] OR “clinical trial, veterinary”[pt] | 3,963,373 |
| 3 | #1 NOT #2 | 5,377 |

**Grey literature search**

A grey literature search was conducted to identify the most recent abstracts, posters, and podium presentations that may not have been indexed in the medical literature databases. These searches were limited to the last two and a half years (2021–2023), to capture the most recent unpublished or ongoing trials. The search covered the following conferences:

- American Society of Tropical Medicine & Hygiene (ASTMH) Annual Meeting
- International Conference on Clinical Microbiology and Infectious Disease Epidemiology (ICCMIDE)
- Conference of the International Society of Travel Medicine (CISTM)
- European Congress of Clinical Microbiology & Infectious Diseases (ECCMID)
- Northern European Conference on Travel Medicine (NECTM)
- International Conference on Tropical Medicine and Infectious Diseases (ICTROMI)
- International Society for Pharmacoeconomics and Outcomes Research (ISPOR): ISPOR Europe, ISPOR-FDA, ISPOR Asia Pacific, ISPOR Latin America, ISPOR Warsaw, ISPOR Dubai

We also conducted bibliographic searches of identified key systematic reviews and meta-analyses (including network meta-analyses), to ensure that the initial searches captured all relevant clinical studies.

**Eligibility criteria**

The eligibility criteria for the search were designed following the Population, Intervention, Comparator, Outcomes, Study (PICOS) type framework. Studies were included if they reported outcomes related to the clinical manifestation of CHIKV, such as the frequency or the severity of the disease in patients with confirmed CHIKV. In addition, we included articles reporting outcomes related to the risk factors associated with its manifestation and collected them for an increasingly detailed filtering process. No limitations were set on the type of intervention and comparator. Studies were excluded if they were in vitro or preclinical studies, reviews, commentaries, letters, or editorials. Moreover, the search was limited to studies in the English language. The exhaustive list of inclusion and exclusion criteria according to the PICOS framework used for the search can be found in Table 3.

Table 3. PICOS framework

| Category | Inclusion criteria | Exclusion criteria |
| --- | --- | --- |
| Population (P) | - Patients infected with CHIKV (including mixed populations) - Healthy volunteers receiving a CHIKV-targeting intervention | None |
| Intervention (I) | Any or no intervention | None |
| Comparators (C) | Any | None |
| Outcomes (O)  (tentative list, not exhaustive) | - Incidence symptomatic/symptomatic disease - Severity and duration of symptoms - Incidence and duration of chronic disease - Chronic resolution rate - Hospitalization frequency - Mortality rate - Efficacy and safety concerns of interventions targeting CHIKV | None |
| Study design (S) | - Randomized (and non-randomized) clinical trial - Systematic reviews and meta-analyses **^a^** - Observational studies - Real-world evidence studies | - In vitro studies - Preclinical studies - Reviews - Comments, letters, and editorials - Case studies - Case series |
| Language | English language | None |
| Time limit | No restriction | None |
| Country | No restriction | None |
| Note: If it is unclear whether a study meets any criterion during the Level 1 screening process, the study will be progressed to full-text screening to confirm its inclusion in the review.  ^a^ Systematic reviews and meta-analyses will be included at Level 1 screening, used for the identification of primary studies, and then excluded at phase 2 screening. | | |
