# Supplementary material for: Clinical outcomes of chikungunya: A systematic literature review and meta-analysis
Source: PLoS Negl Trop Dis. 2024 Jun 7;18(6):e0012254. doi: 10.1371/journal.pntd.0012254 (PMC11189168; doi:10.1371/journal.pntd.0012254)
Supplement: S2 Text — Modified Downs & Black checklist and the NIH quality assessment tool. (DOCX) [file pntd.0012254.s002.docx]

**S2 Text. Quality assessment tools.**

A quality assessment for all included studies was performed using the Downs and Black checklist for assessing the risk of bias [1] or the NIH quality assessment tool for observational cohort and cross-sectional studies [2]. An example of these tools is presented in Table S2.1 and 2. The quality assessment was performed by one independent researcher.

The quality of the interventional studies was evaluated using a modified version of the Downs and Black checklist (i.e., excluding Question 27) [1]. Questions 1 through 10 in the checklist can be answered with ‘yes’ or ‘no’, except for Question 5, which can be answered with ‘yes’, ‘partially’, or ‘no’. Questions 11 through 26 can be answered with ‘yes’, ‘no’, or ‘unable to determine’. Each item is positively stated (i.e., it presents what is desired) and given a score, with a possible total score of 27 for the modified version. A higher score indicates a higher quality study.

The quality of the observational studies was evaluated using the NIH quality assessment tool for observational cohort and cross-sectional studies [2]. Questions 1 through 14 in the checklist can be answered with ‘yes’, ‘no’, or ‘other (cannot determine [CD]; not applicable [NA]; not reported [NR])’. After answering the questions, a quality rating was performed. The articles were rated as good, fair, or poor, according to the reported answers. A score of 11-14 indicated ‘good’ quality, a score of 5-10 indicated ‘fair’ quality, and a score of 0-4 indicated ‘poor’ quality. If a question was answered with ‘other (CD, NR, NA)’, the answer was considered as ‘yes’ if it did not affect the quality of the article.

Table S2.1. Downs and Black quality assessment checklist

| **Question No.** | **Question** |
| --- | --- |
| **1** | Is the hypothesis/aim/objective of the study clearly described? |
| **2** | Are the main outcomes to be measured clearly described in the introduction or methods section? |
| **3** | Are the characteristics of the patients included in the study clearly described? |
| **4** | Are the interventions of interest clearly described? |
| **5** | Are the distributions of principal confounders in each group of patients to be compared clearly described? |
| **6** | Are the main findings of the study clearly described? |
| **7** | Does the study provide estimates of the random variability in the data for the main outcomes? |
| **8** | Have all important AE that may be a consequence of the intervention been reported? |
| **9** | Have the characteristics of patients lost to follow-up been described? |
| **10** | Have actual probability values been reported (e.g., 0.035 rather than <0.05) for the main outcomes except where the probability value is less than 0.001? |
| **11** | Were the subjects asked to participate in the study representative of the entire population from which they were recruited? |
| **12** | Were those subjects who were prepared to participate representative of the entire population from which they were recruited? |
| **13** | Were the staff, places, and facilities where the patients were treated representative of the treatment the majority of patients receive? |
| **14** | Was an attempt made to blind study subjects to the intervention they have received? |
| **15** | Was an attempt made to blind those measuring the main outcomes of the intervention? |
| **16** | If any of the results of the study were based on ‘data dredging’, was this made clear? |
| **17** | In trials and cohort studies, do the analyses adjust for different lengths of follow-up of patients, or in case-control studies, is the time period between the intervention and outcome the same for cases and controls? |
| **18** | Were the statistical tests used to assess the main outcomes appropriate? |
| **19** | Was in compliance with the intervention(s) reliable? |
| **20** | Were the main outcome measures used accurate (valid and reliable)? |
| **21** | Were the patients in different intervention groups (trials and cohort studies) or were the cases and controls (case-control studies) recruited from the same population? |
| **22** | Were study subjects in different intervention groups (trials and cohort studies) or were the cases and controls (case-control studies) recruited over the same period of time? |
| **23** | Were study subjects randomized to intervention groups? |
| **24** | Was the randomized intervention assignment concealed from both patients and health care staff until recruitment was complete and irrevocable? |
| **25** | Was there an adequate adjustment for confounding in the analyses from which the main findings were drawn? |
| **26** | Were losses of patients to follow-up taken into account? |

Table S2.2. NIH quality assessment tool for observational cohort and cross-sectional studies

| Criteria | Yes | No | Other |
| --- | --- | --- | --- |
| 1. Was the research question or objective in this paper clearly stated? |  |  |  |
| 2. Was the study population clearly specified and defined? |  |  |  |
| 3. Was the participation rate of eligible persons at least 50%? |  |  |  |
| 4. Were all the subjects selected or recruited from the same or similar populations (including the same time period)? Were inclusion and exclusion criteria for being in the study prespecified and applied uniformly to all participants? |  |  |  |
| 5. Was a sample size justification, power description, or variance and effect estimates provided? |  |  |  |
| 6. For the analyses in this paper, were the exposure(s) of interest measured prior to the outcome(s) being measured? |  |  |  |
| 7. Was the timeframe sufficient so that one could reasonably expect to see an association between exposure and outcome if it existed? |  |  |  |
| 8. For exposures that can vary in amount or level, did the study examine different levels of the exposure as related to the outcome (e.g., categories of exposure, or exposure measured as continuous variable)? |  |  |  |
| 9. Were the exposure measures (independent variables) clearly defined, valid, reliable, and implemented consistently across all study participants? |  |  |  |
| 10. Was the exposure(s) assessed more than once over time? |  |  |  |
| 11. Were the outcome measures (dependent variables) clearly defined, valid, reliable, and implemented consistently across all study participants? |  |  |  |
| 12. Were the outcome assessors blinded to the exposure status of participants? |  |  |  |
| 13. Was loss to follow-up after baseline 20% or less? |  |  |  |
| 14. Were key potential confounding variables measured and adjusted statistically for their impact on the relationship between exposure(s) and outcome(s)? |  |  |  |

**References**

1. Downs, S. H., & Black, N. The feasibility of creating a checklist for the assessment of the methodological quality both of randomised and non-randomised studies of health care interventions. Journal of Epidemiology & Community Health*.* 1998; 52(6): 377-384.

2. NIH National Heart, Lung and Blood Institute. Study quality assessment tools [cited 2024, February 7]. Available from: https://www.nhlbi.nih.gov/health-topics/study-quality-assessment-tools
