# Supplementary material for: Clinical outcomes of chikungunya: A systematic literature review and meta-analysis
Source: PLoS Negl Trop Dis. 2024 Jun 7;18(6):e0012254. doi: 10.1371/journal.pntd.0012254 (PMC11189168; doi:10.1371/journal.pntd.0012254)

Forest Plots

*Target Population*

**Hospitalization**
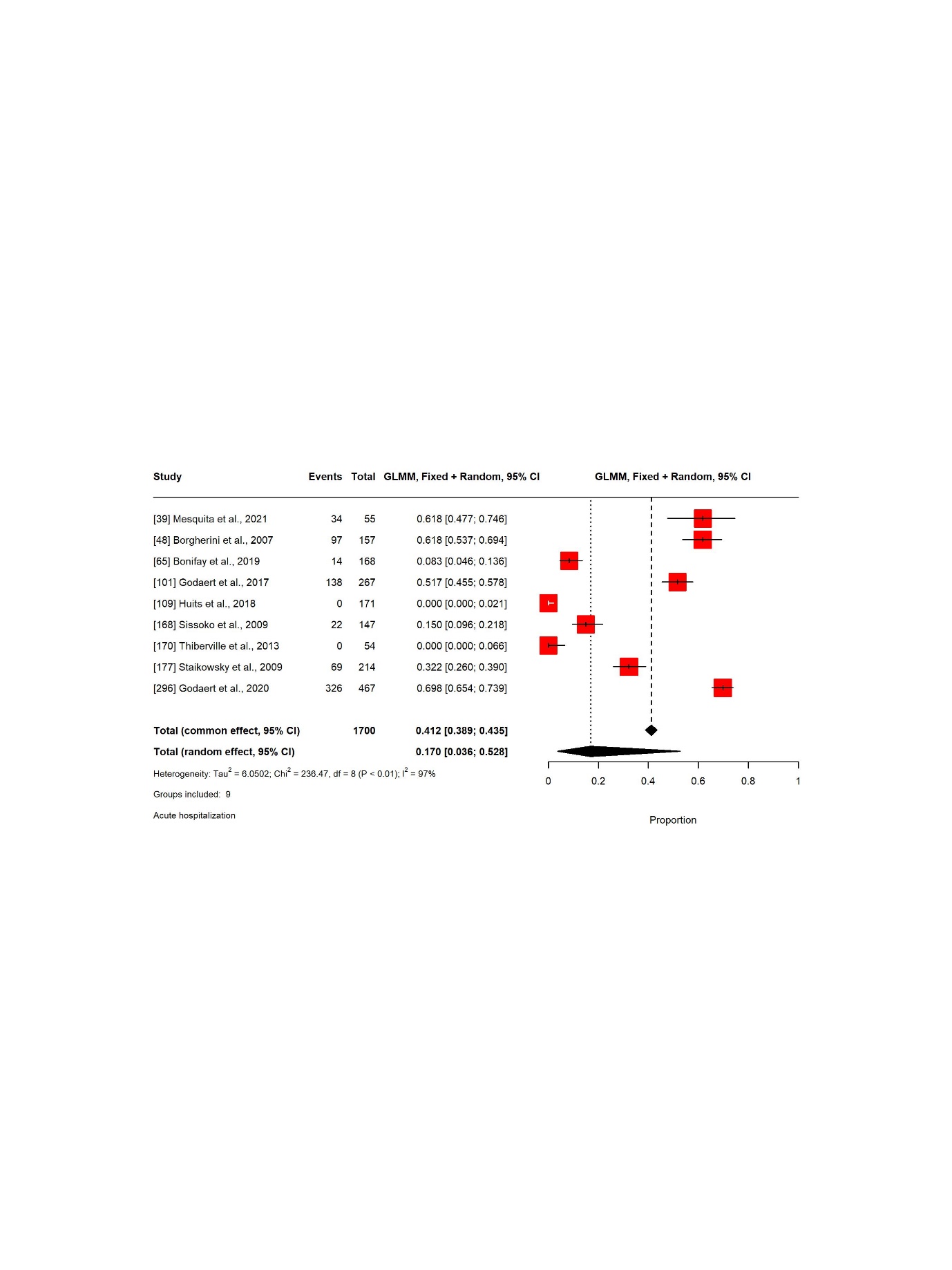


**
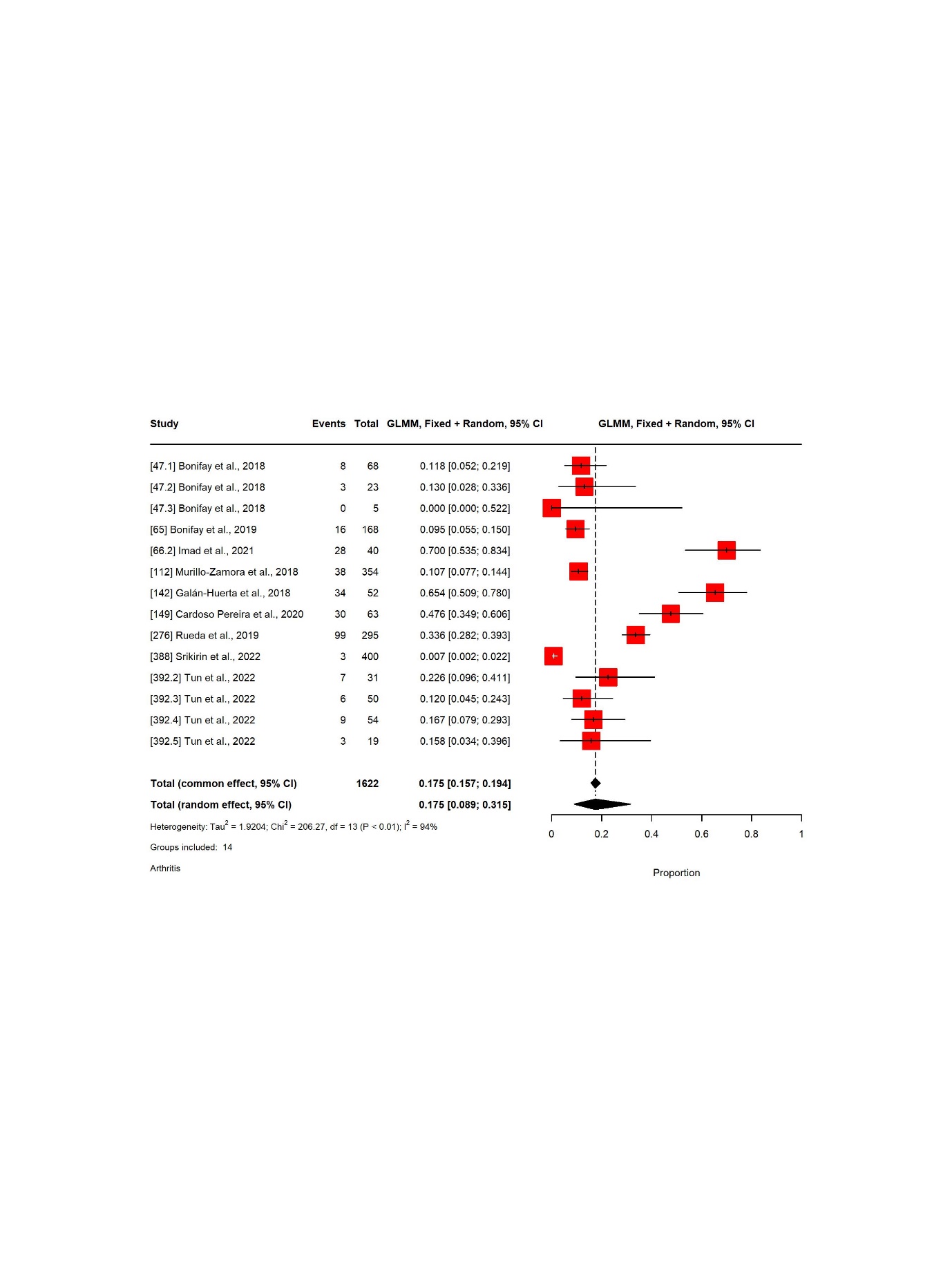
Arthritis**

**Arthralgia**


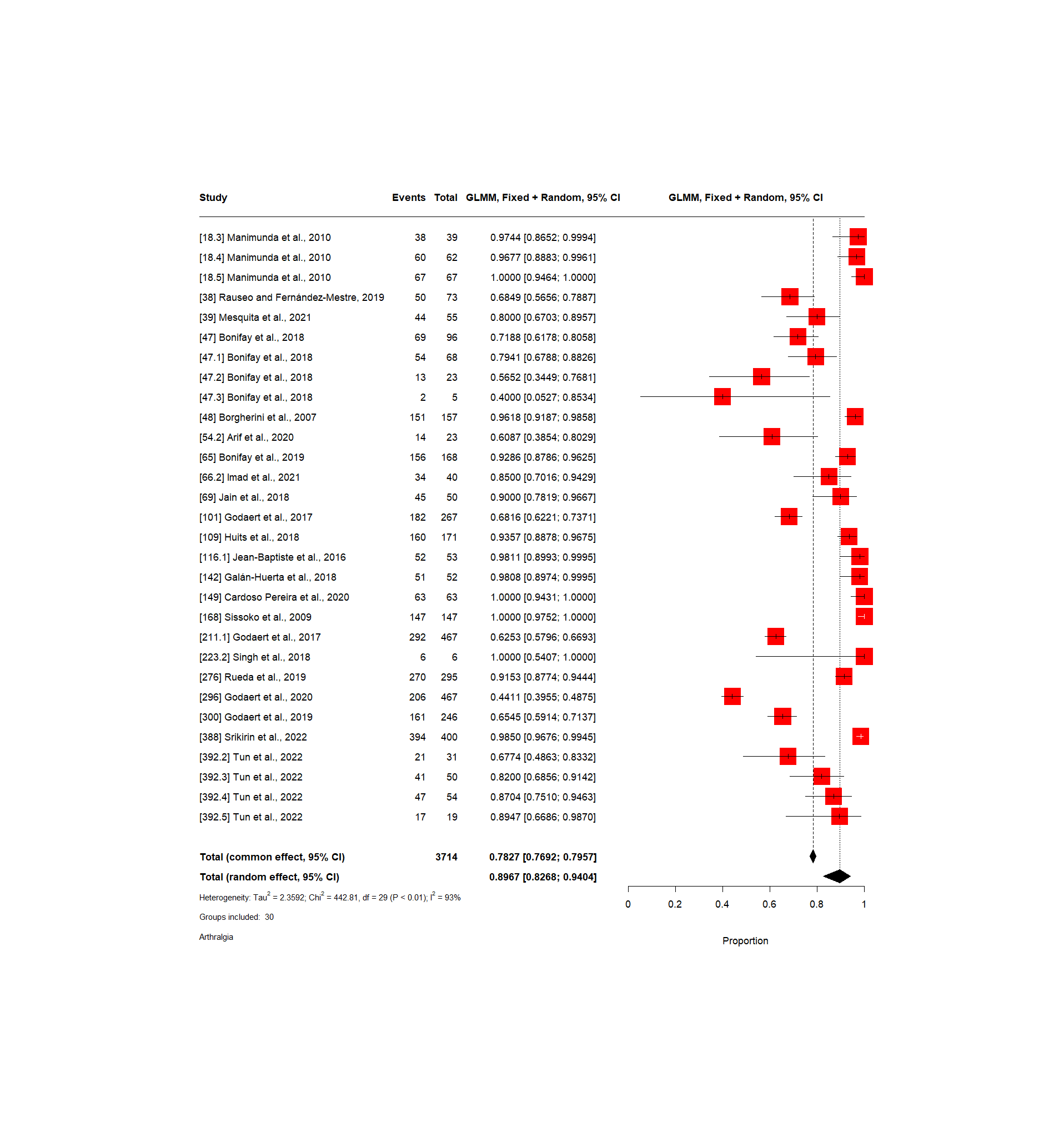


**Fever**
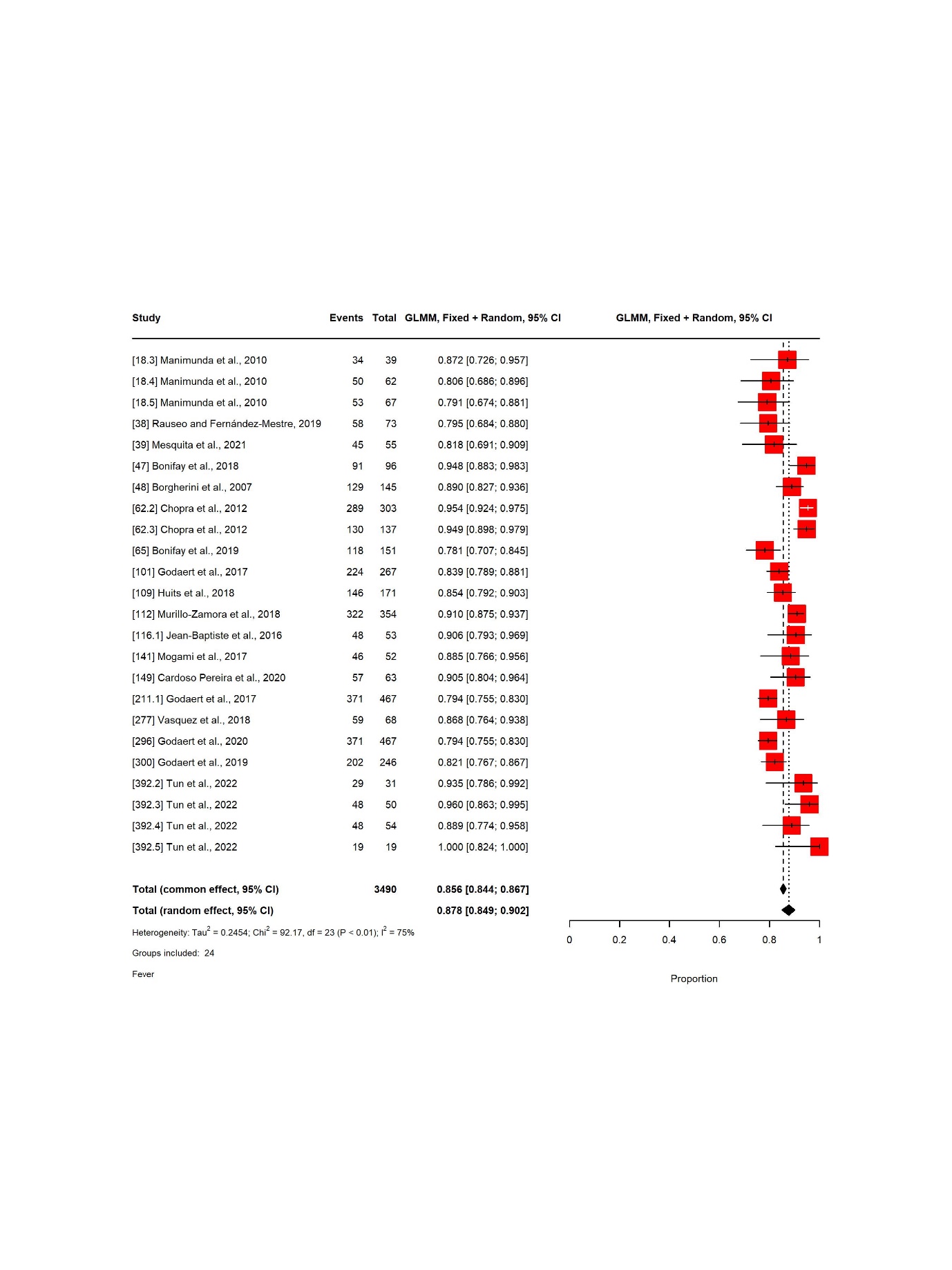


**Fatigue**
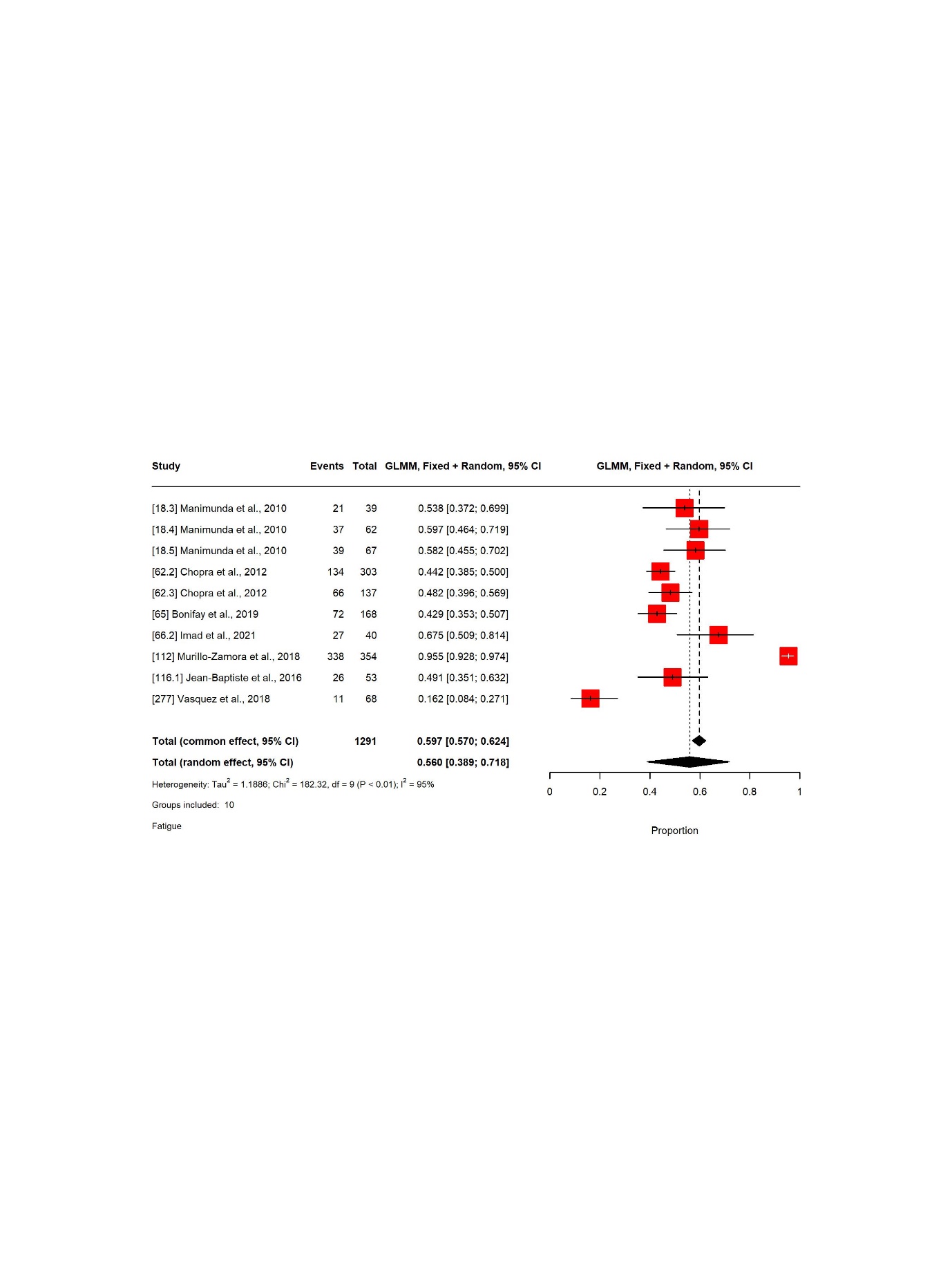


**Headache**
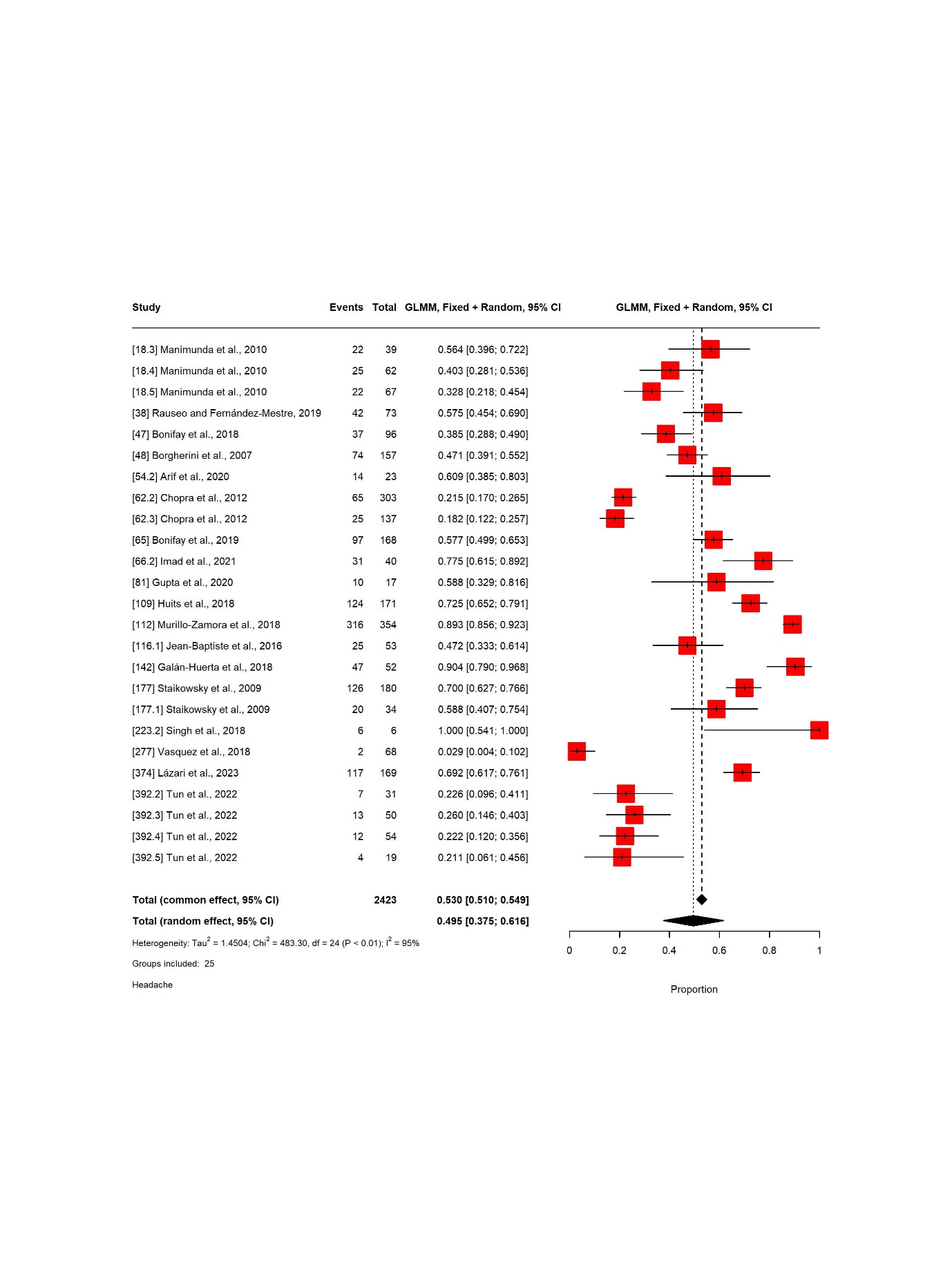


**Joint Swelling**
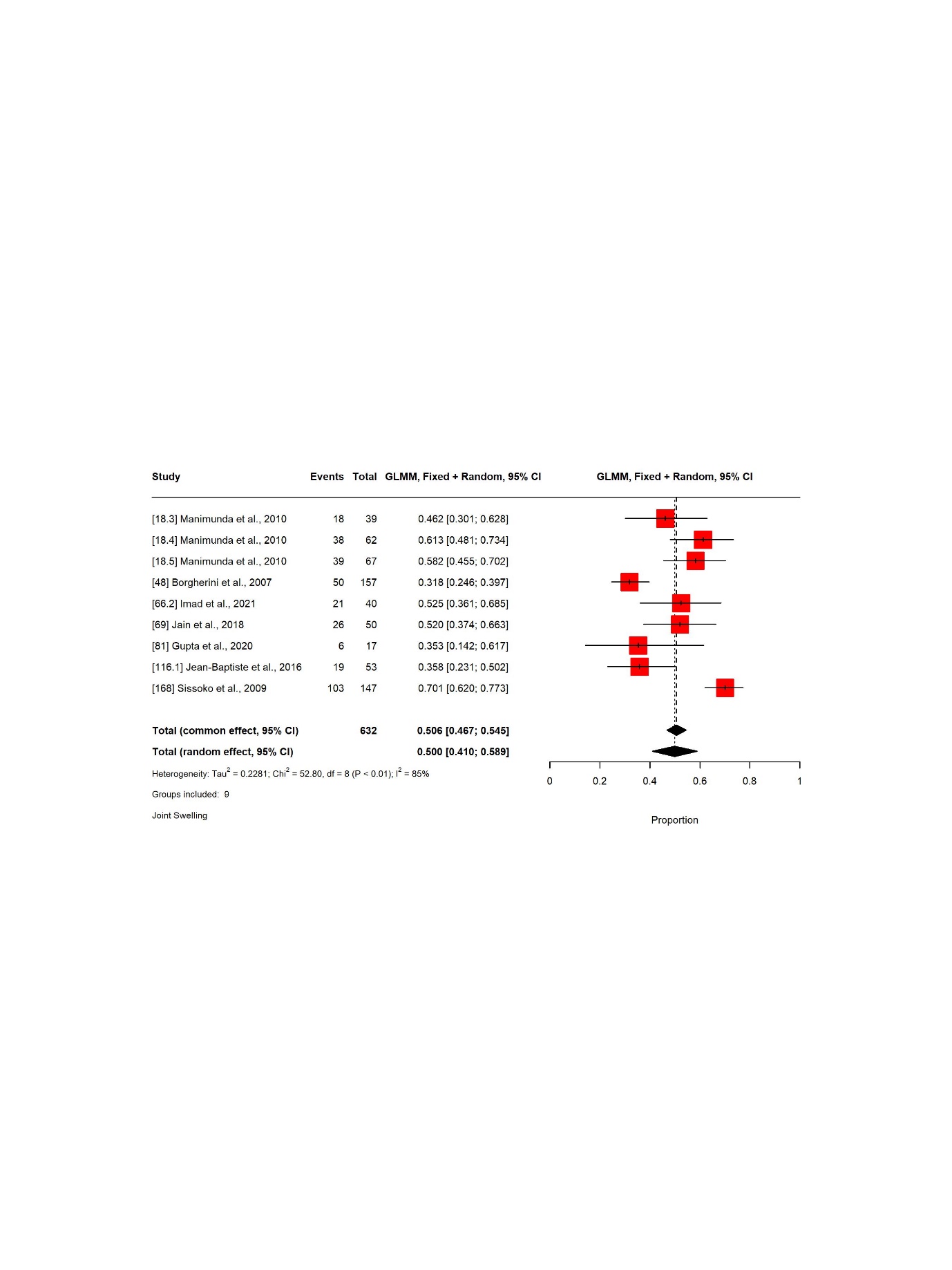


**Myalgia**
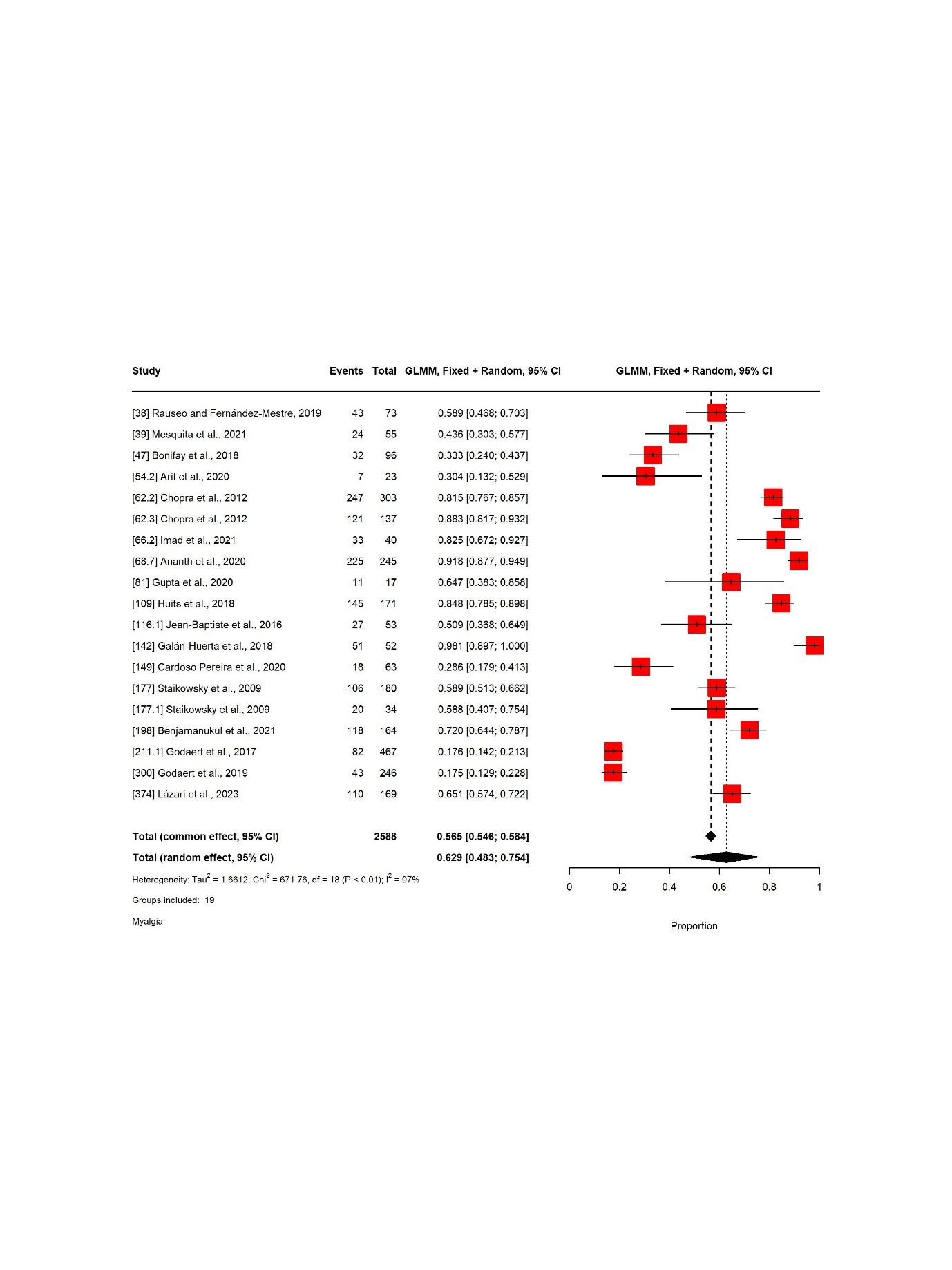


**Nausea**
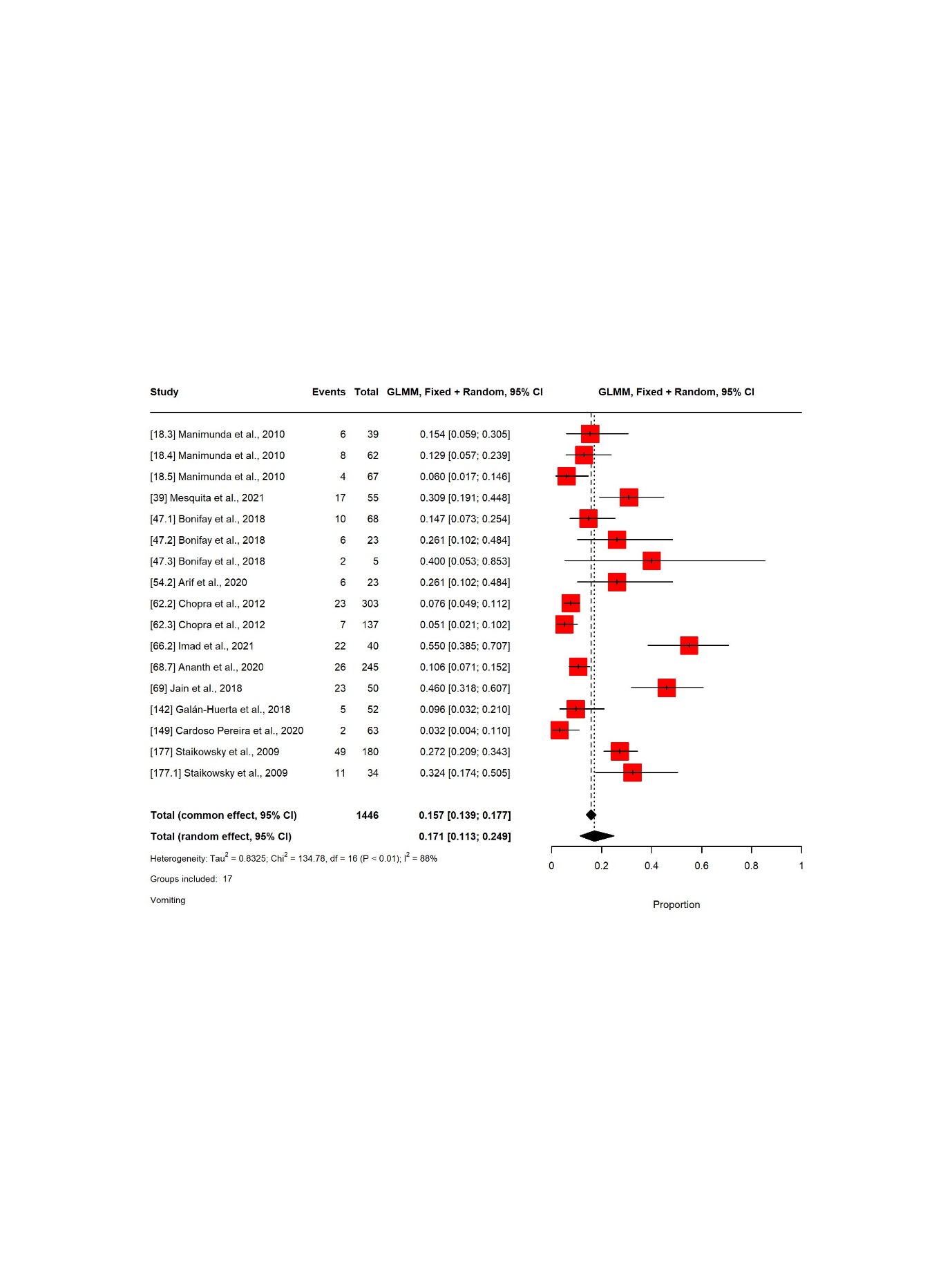


**Rash**
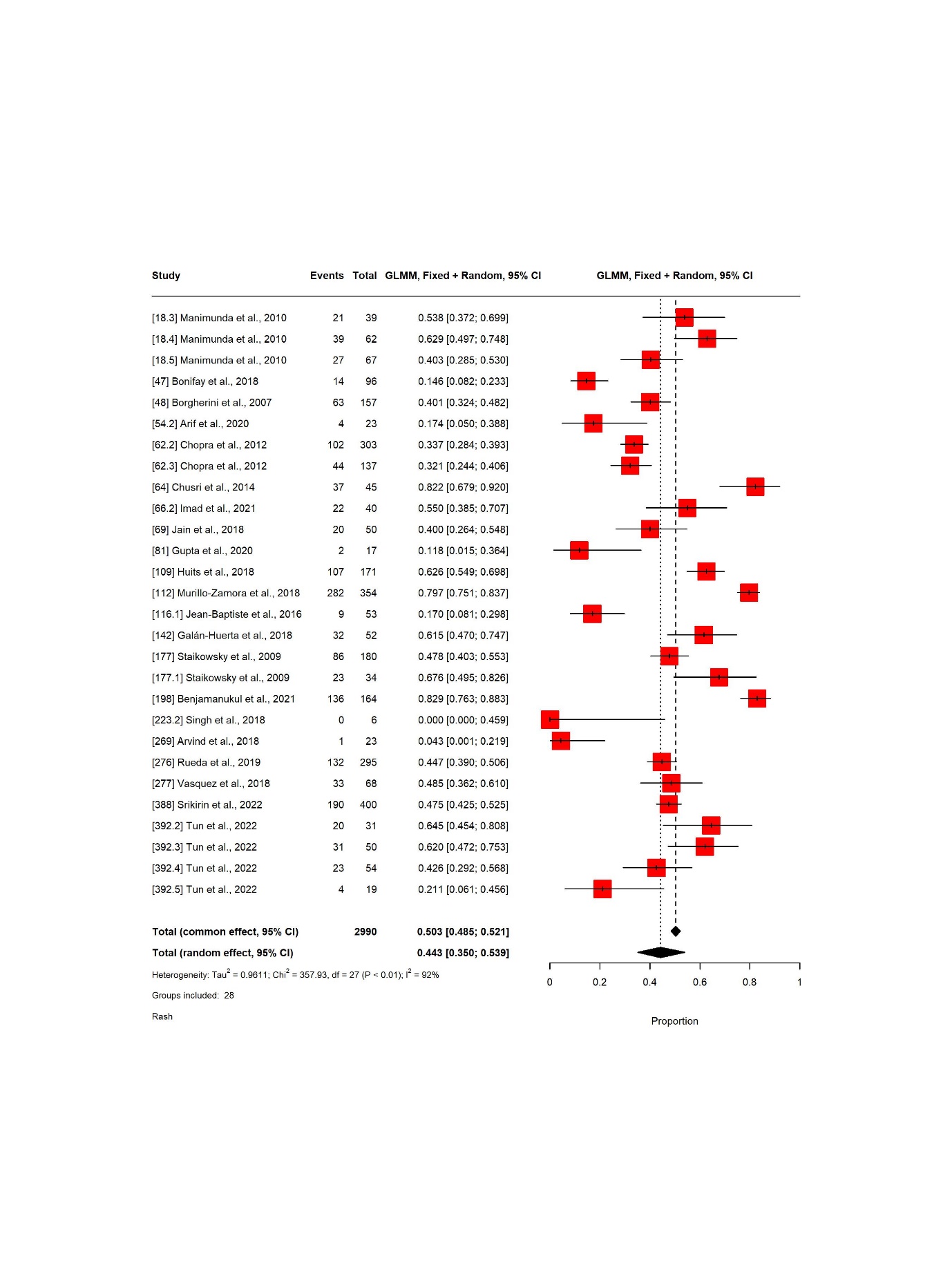


**Vomiting**
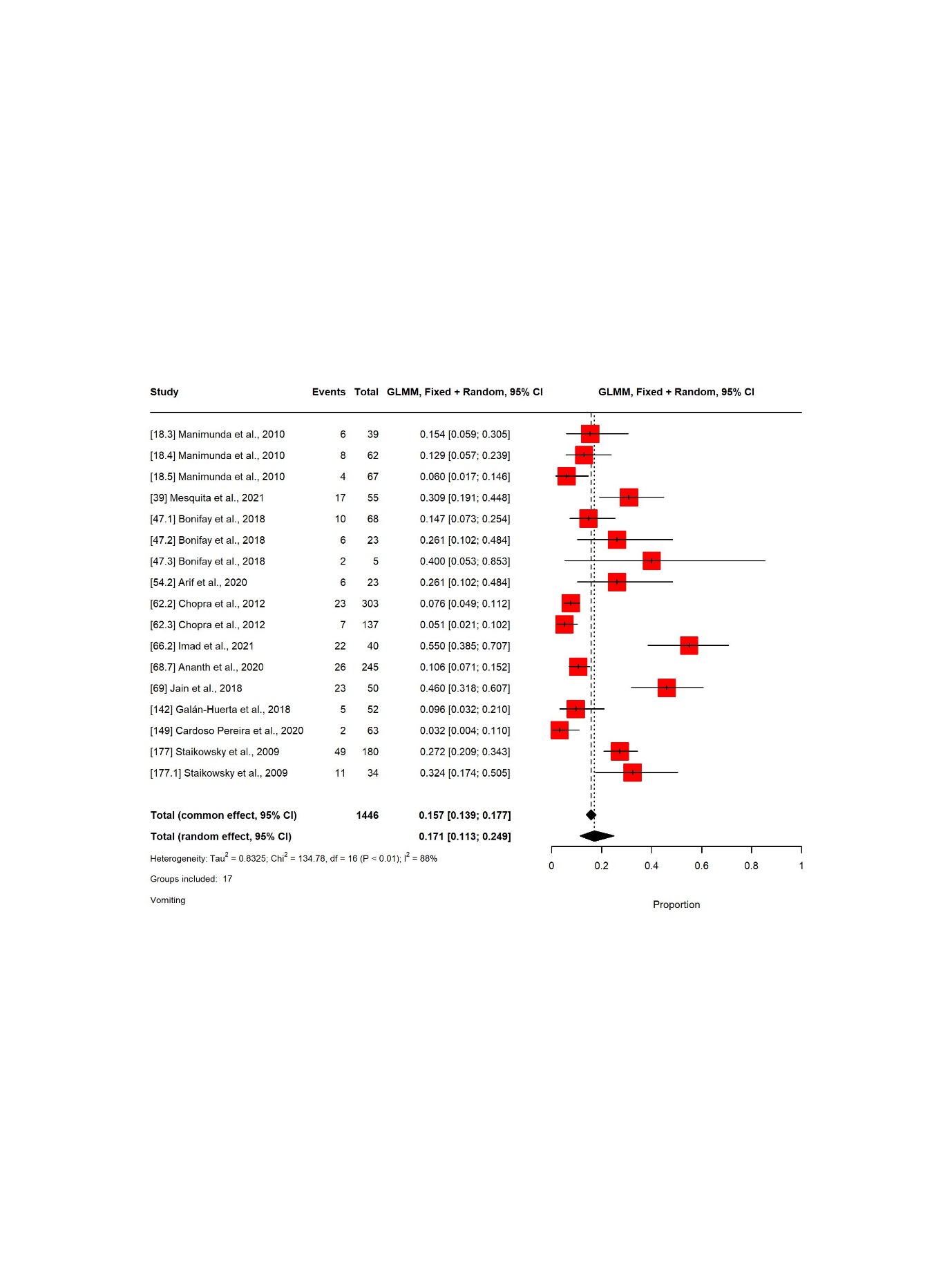


*General Population*

**Chronic Rate**
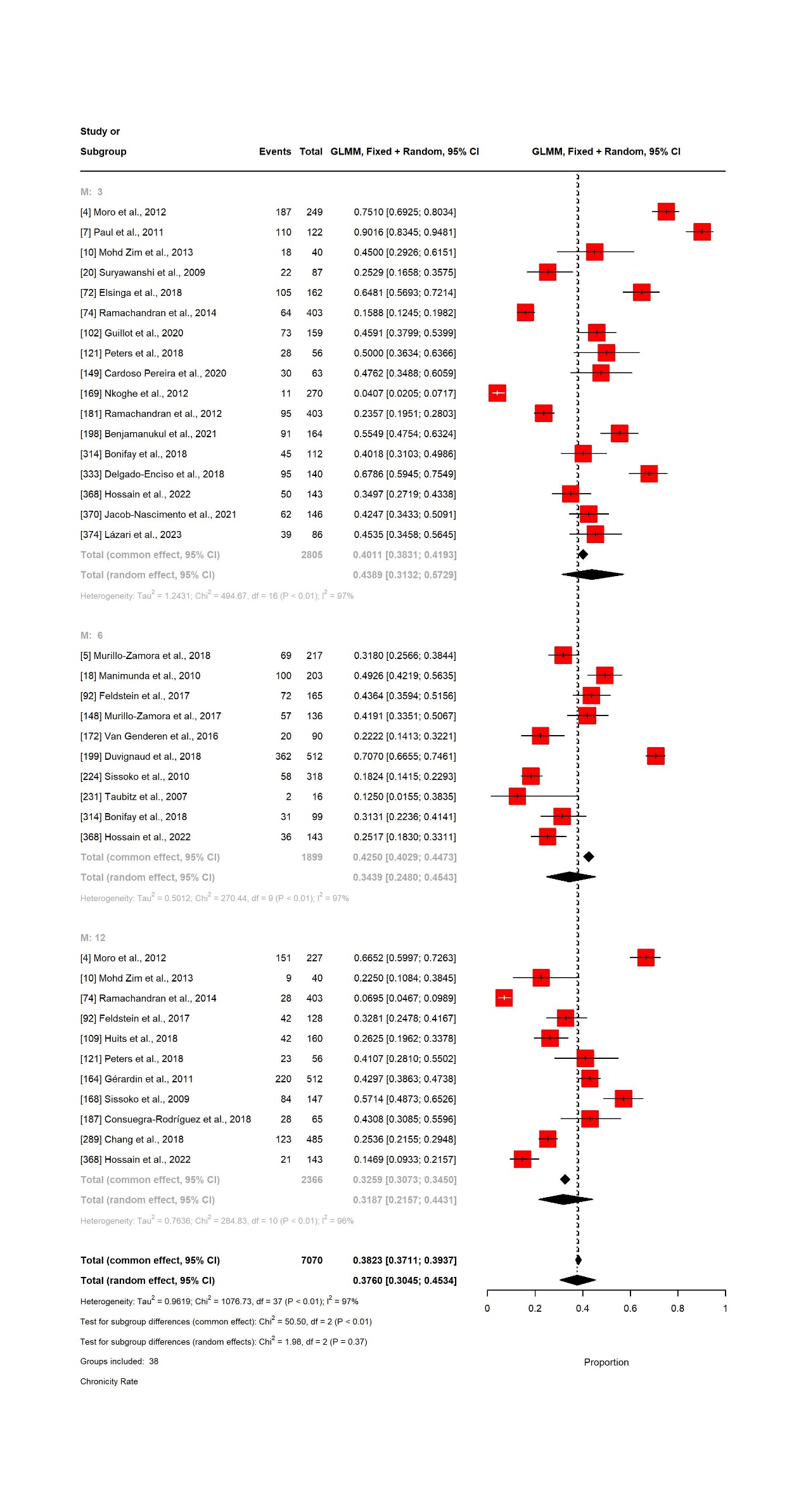


**Mortality Rate**
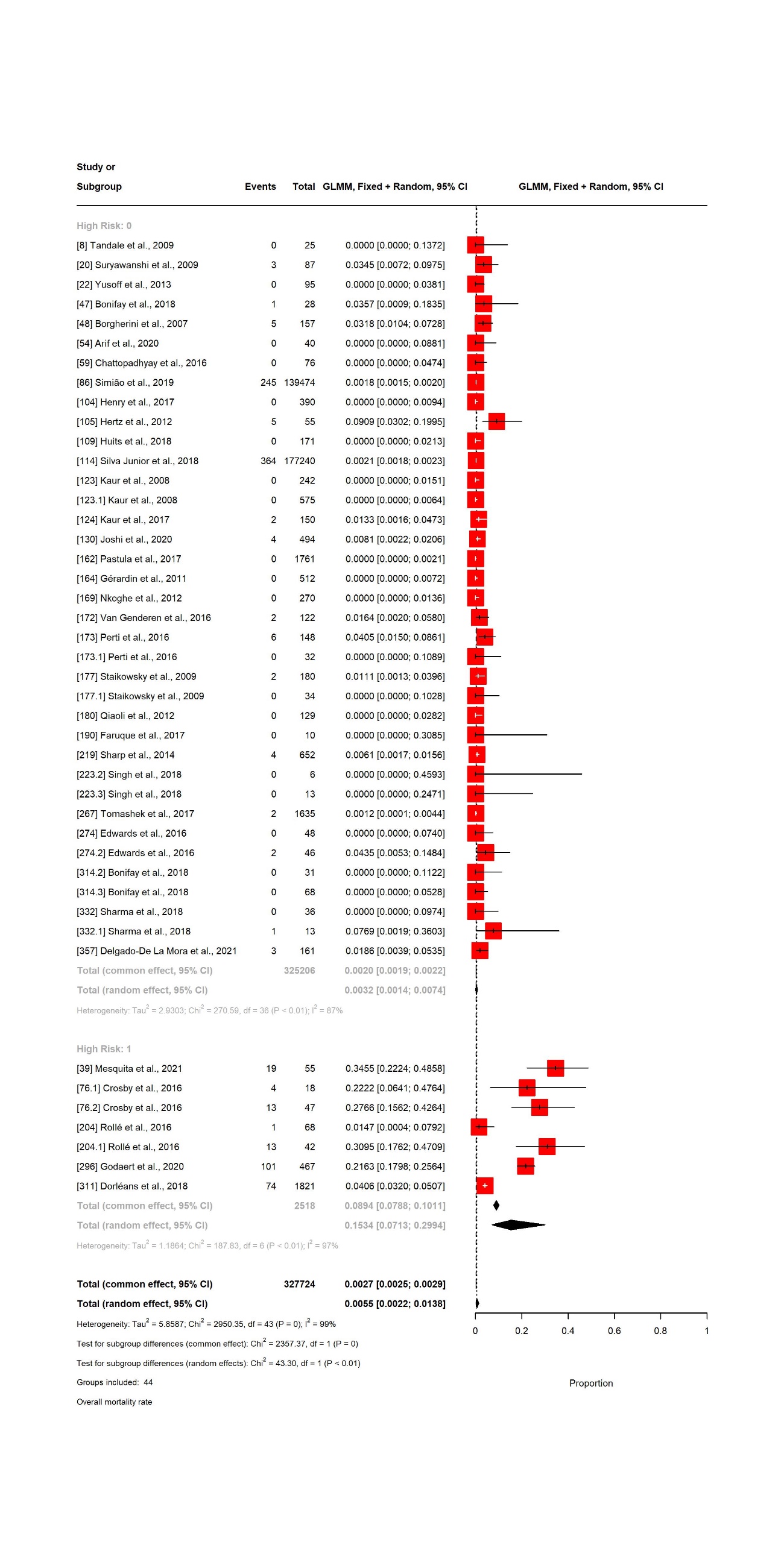


**Symptomatic Rate**
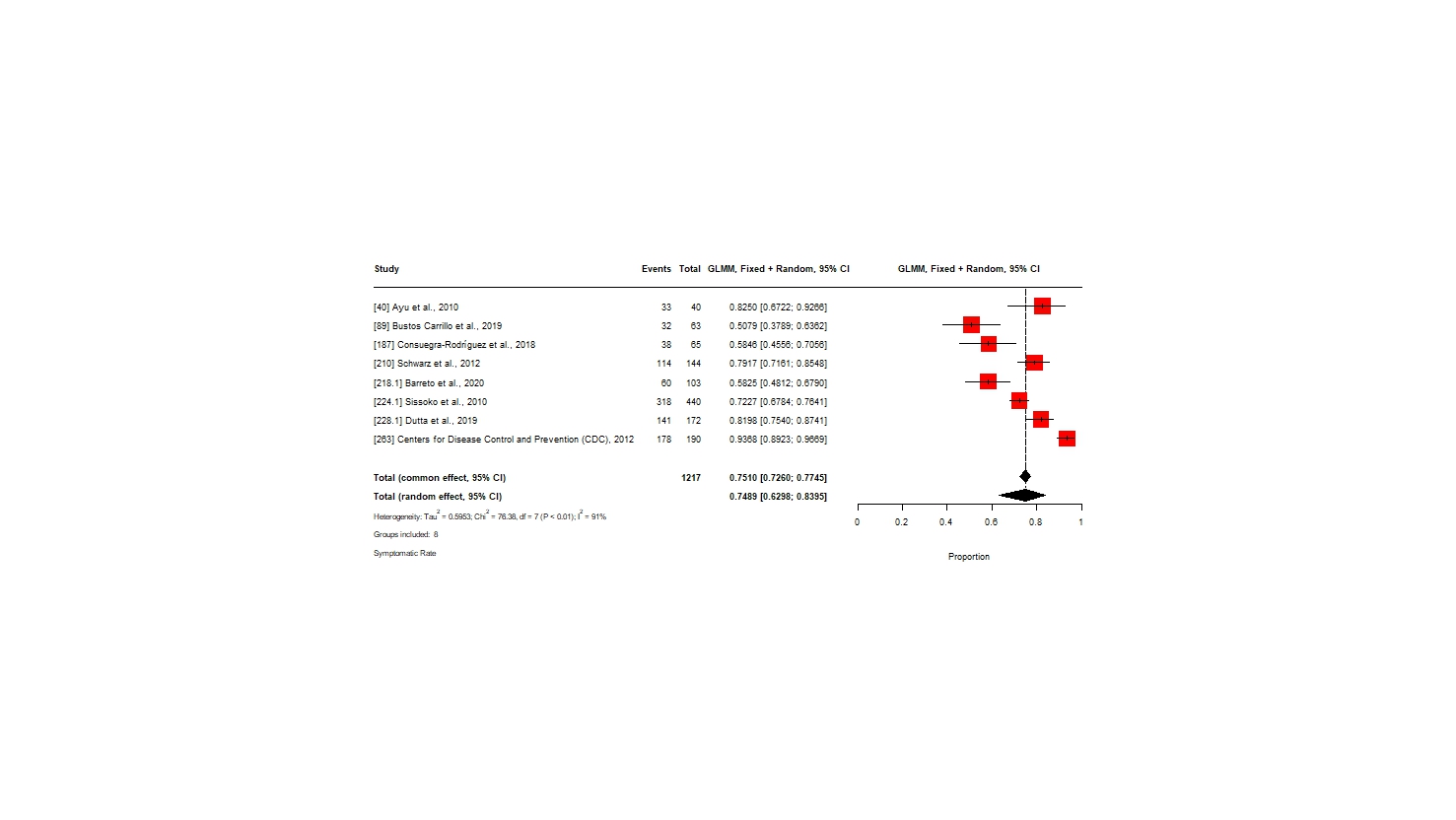

Supplement: S1 Fig — (DOCX) [file pntd.0012254.s006.docx]
