# Supplementary material for: Clinical outcomes of chikungunya: A systematic literature review and meta-analysis
Source: PLoS Negl Trop Dis. 2024 Jun 7;18(6):e0012254. doi: 10.1371/journal.pntd.0012254 (PMC11189168; doi:10.1371/journal.pntd.0012254)

**S2 Fig. Influential case and outlier analyses with Baujat plots**

When one or more studies report extreme effect size values, this is considered an ‘outlying’ study. These studies could distort the outcomes generated through the meta-analyses, especially when the number of included studies is low. Studies can also have a strong impact on the direction of the results when they are not considered outliers. These studies are called influential studies, and their presence and impact were also investigated. Influential case analysis was done by removing one study at a time form each meta-analysis to quantify the effect of any single study on the results. The influence of a study on the pooled results is illustrated with Baujat plots. Influential case analysis and Baujat plots were done for all meta-analysis report, but no outlier was removed from any analysis.

*Target Population*

**Hospitalization**

Figure 1. The sensitivity analyses indicated three outliers within the meta-analysis of hospitalization, including the population reported by Bonifay et al. [65]. This particular study included patients who were diagnosed or treated at the hospital, but that did not provide any indications regarding its contribution to the overall heterogeneity. Another population that was identified as an outlier was reported in the study by Sissoko et al. [168]; however, once again, after checking the inclusion criteria and group characteristics, we found no indications as to why this group is an outlier. The last group that was identified as an outlier was reported by Godaert et al. [296]. This population had a mean age of 80.4 years old and medical files were used for data collection, both of which could have influenced the number of hospitalizations. Since all populations fall within the criteria set out for the filter, they were all kept in the analysis.


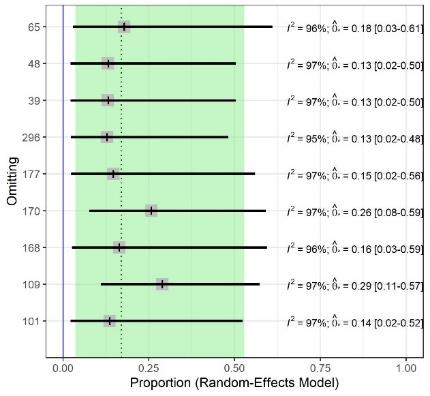

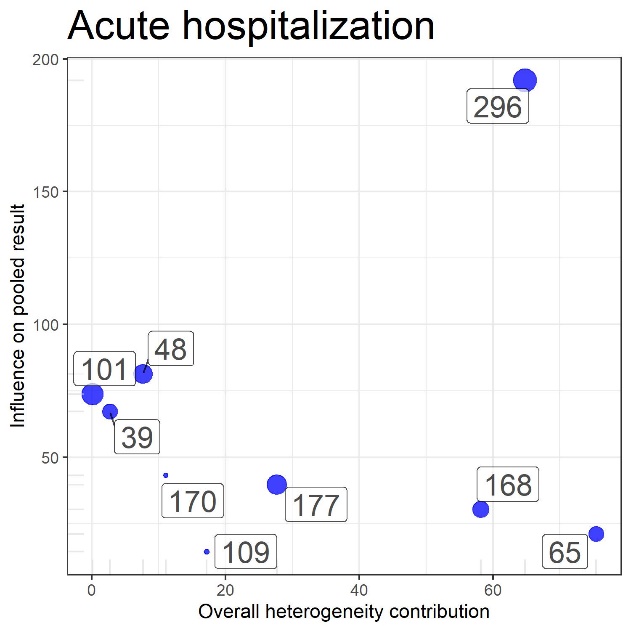


**Arthritis**


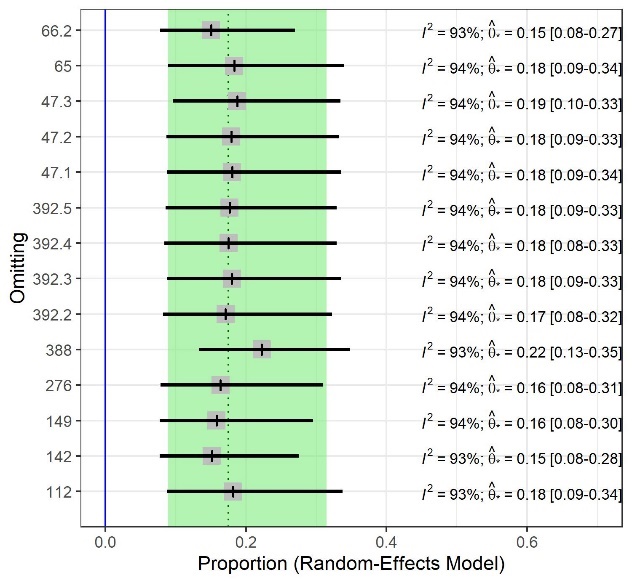

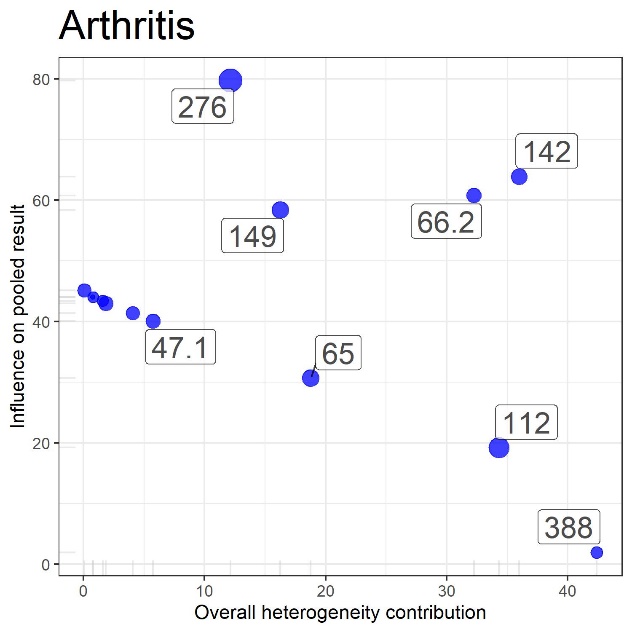


Figure 2. During this analysis, multiple studies stood out as outliers when compared to the pooled estimate. The two most significant outlying populations were reported by Galán-Huerta et al. [142] and Srikirin et al. [388] Both studies were checked for their consistency with the general population description. After a thorough evaluation of the inclusion criteria and characteristics of each group, it was determined that the populations were consistent with the specified general population. Therefore, both studies were included in this analysis.

**Arthralgia**


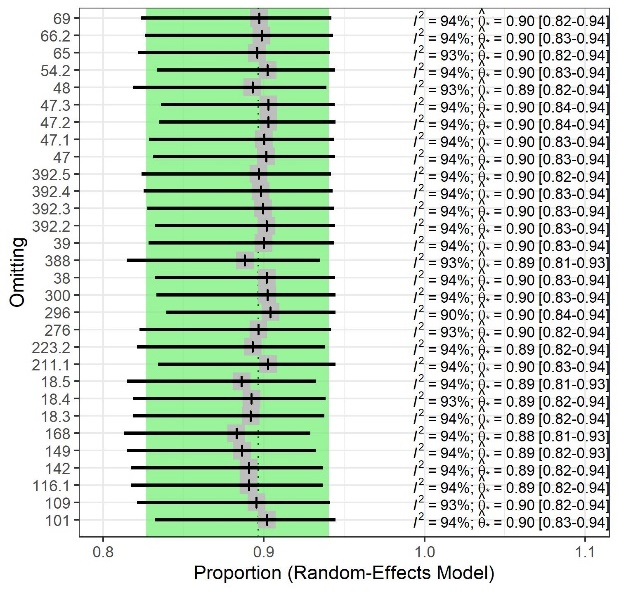

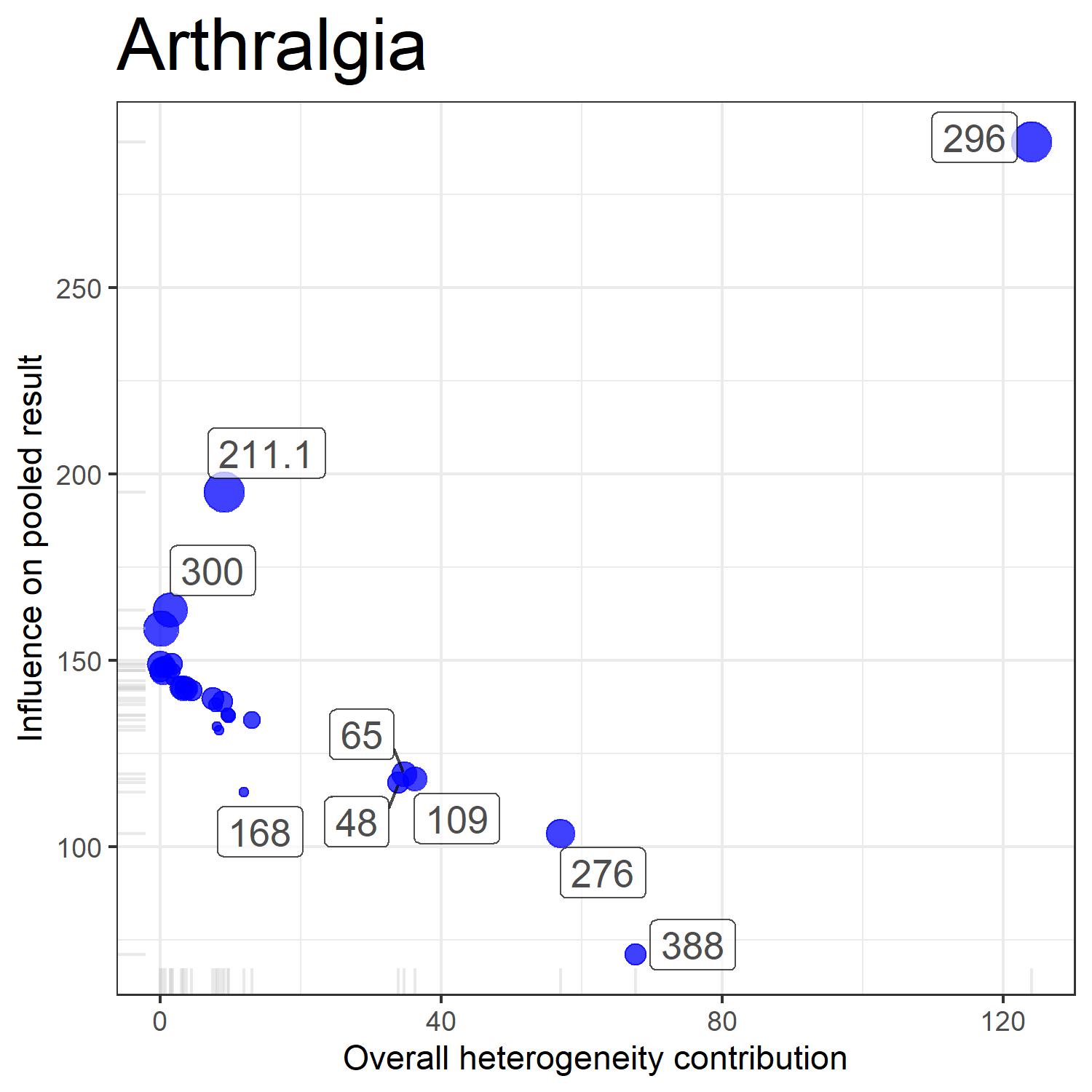


Figure 3. During these analyses, we identified several studies as outliers and, initially, we found the population reported by Godeart et al. [296] in 2020 to be the most influential and outlying. However, upon careful examination of inclusion criteria and characteristics for each group, we concluded that the populations were similar to each other as well as aligned with the specified general population. One notable observation was that the occurrence of arthralgia was consistently lower in groups of individuals aged 65 years or older, compared to other age groups. This was also the population included by Godeart et al. in 2020.

**Fever**

Figure 4. Following the sensitivity analyses, one of the populations reported by Chopra et al. [62.2] was identified as an outlier. However, since there was no indication that this study differed from the general population, we decided to retain it in the meta-analysis.


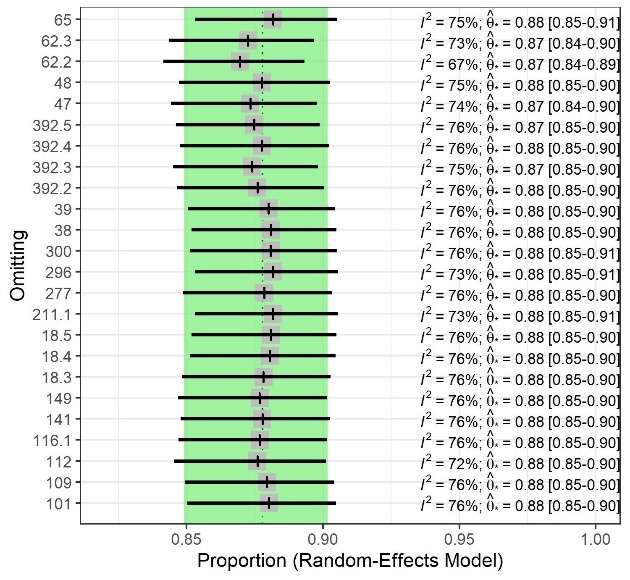

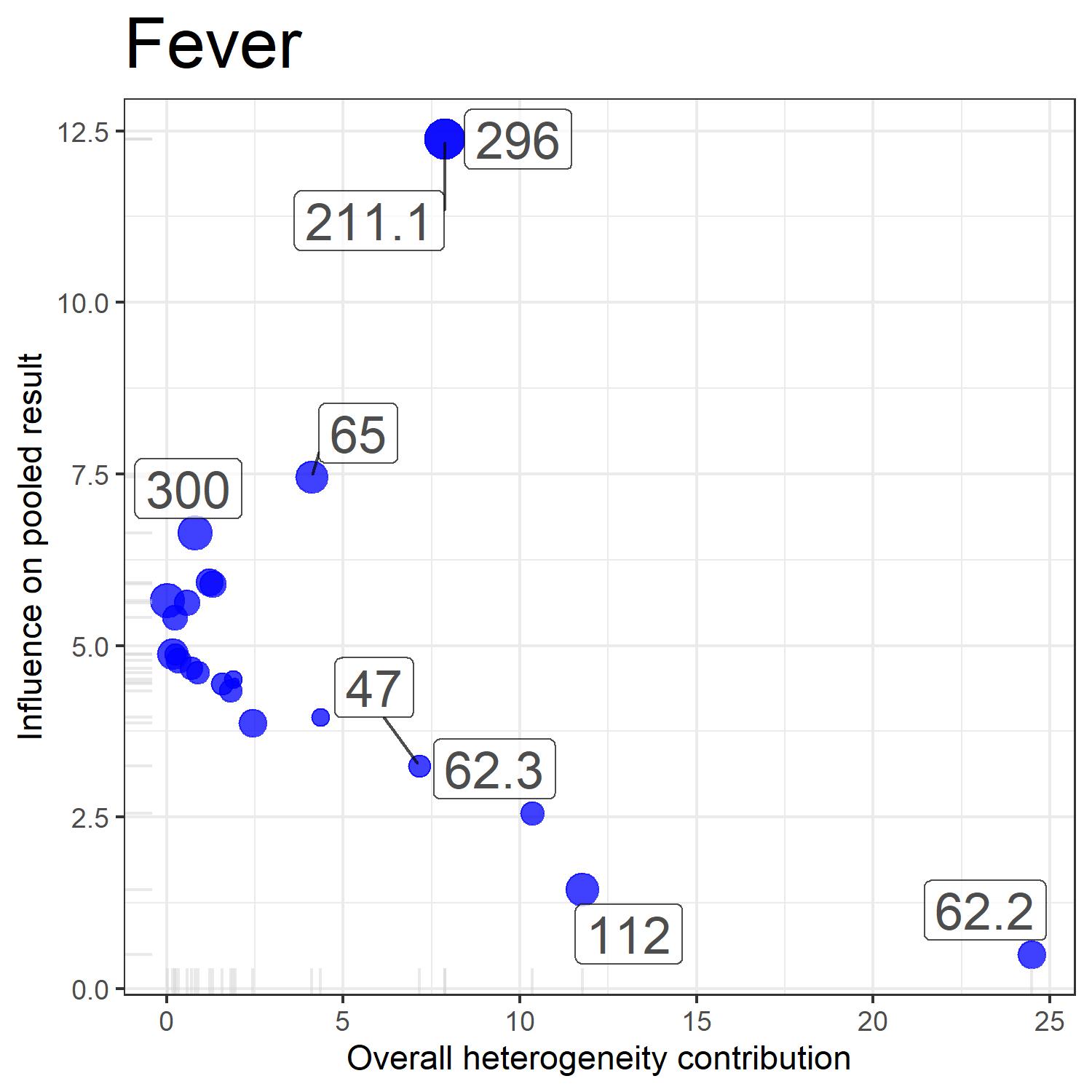


**Fatigue**

Figure 5. During this analysis, we identified the group reported by Murillo-Zamora et al. [112] to be an outlier. As can be seen in the forest plot, their study identified a significantly higher number of patients who experienced fatigue than the other studies. The goal of their study was to screen for depressive mood experienced during acute chikungunya and to evaluate the association of several exposures with the risk of depressive symptoms. However, they describe a population that falls within our general population criteria, without mentioning anything about the population being more at risk for depressive symptoms. Therefore, the study was retained in the meta-analysis for the fatigue endpoint.


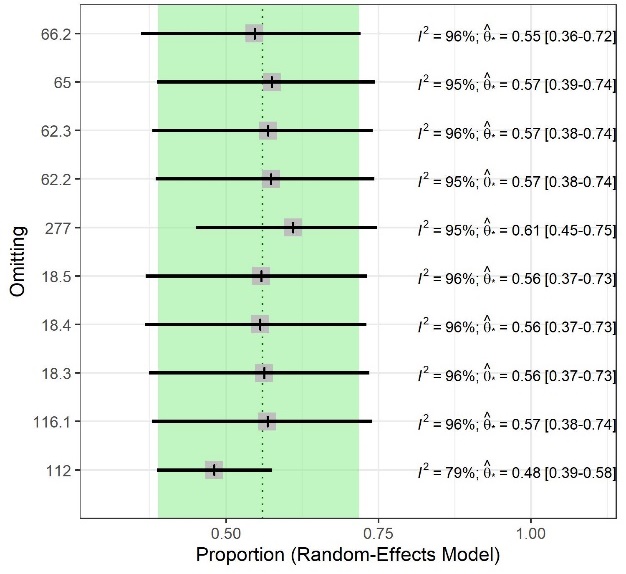

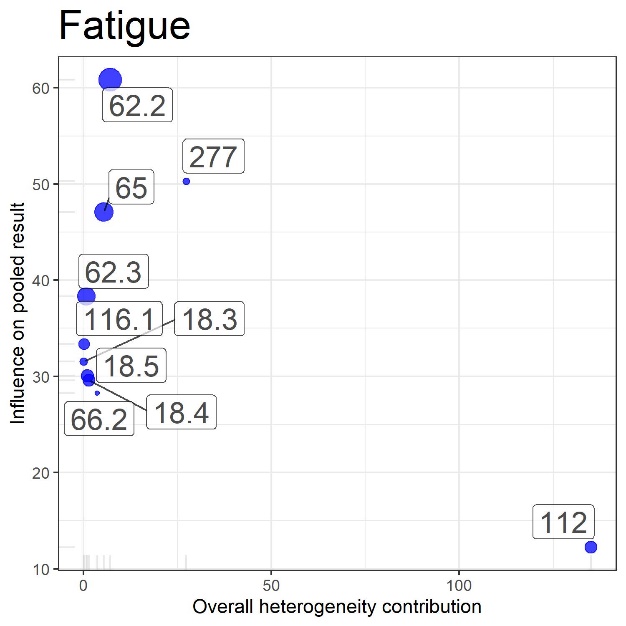


**Headache**

Figure 6. The sensitivity analyses indicated that one of the groups reported by the study of Chopra et al. [62.2] was outlying, as well as the group reported by Murillo-Zamora et al. [112] These groups were previously identified as outliers in the meta-analysis on fever and fatigue, respectively. Both studies were retained for the meta-analysis on headache.


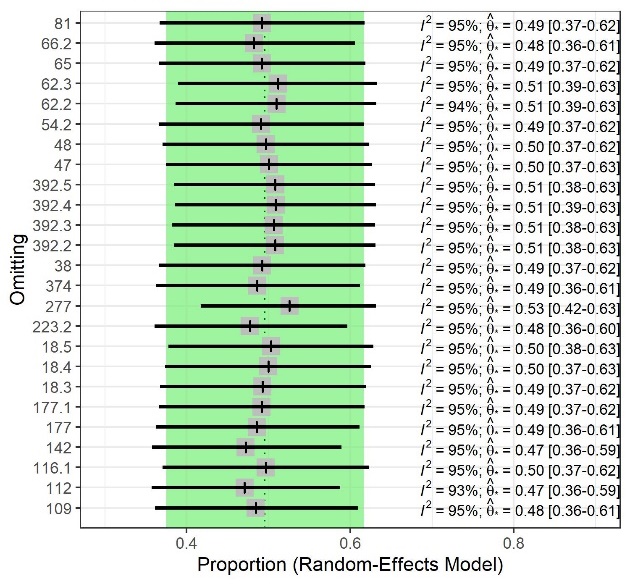

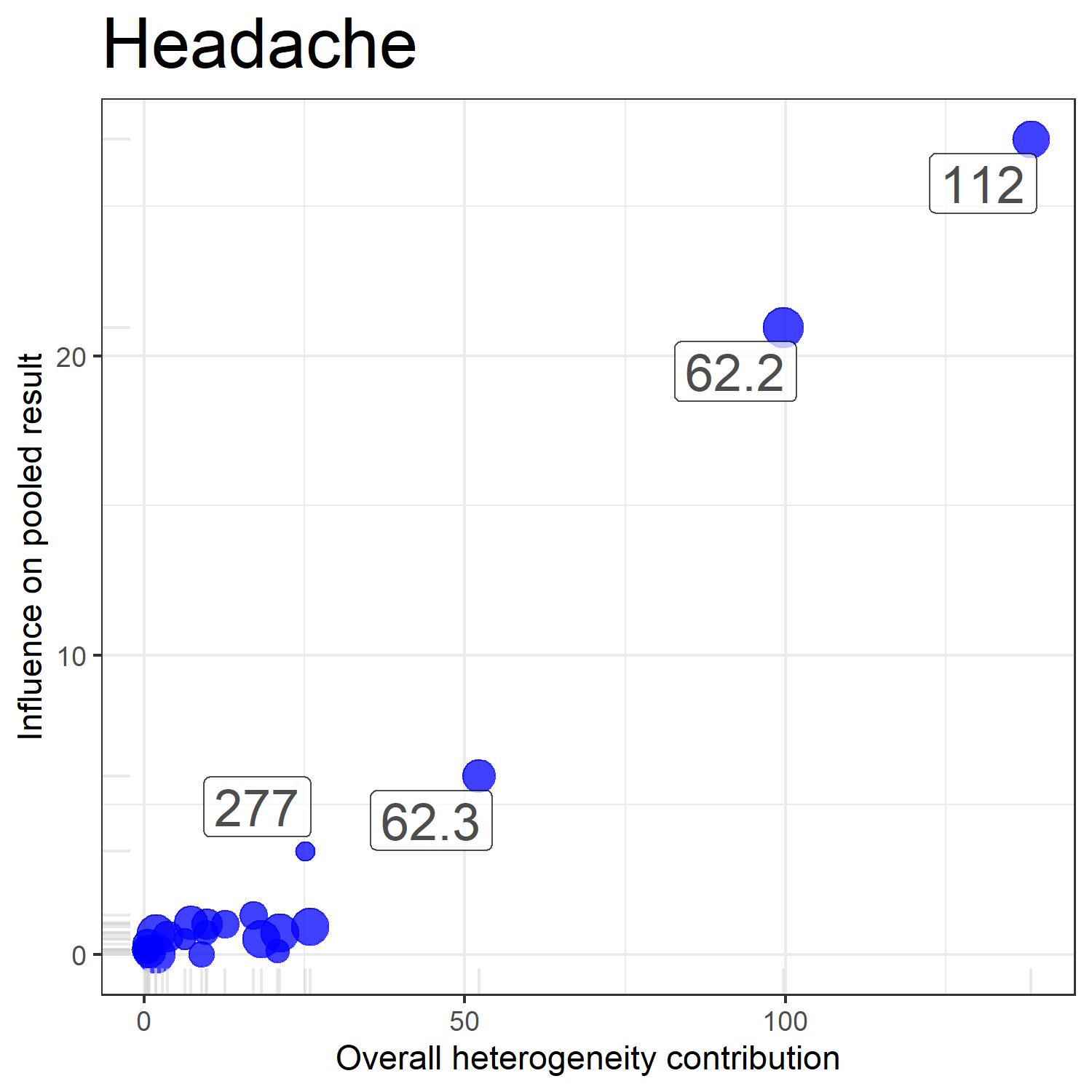


**Joint Pain**

Figure 7. There were three groups identified as outliers in the meta-analysis on joint pain, which were reported by the same study by Bonifay et al. [47] This study includes patients with clinical presentation and confirmed acute chikungunya. We were not able to find indications as to why this study contributes highly to the overall heterogeneity in this meta-analysis. The study was retained in the meta-analysis for joint pain.


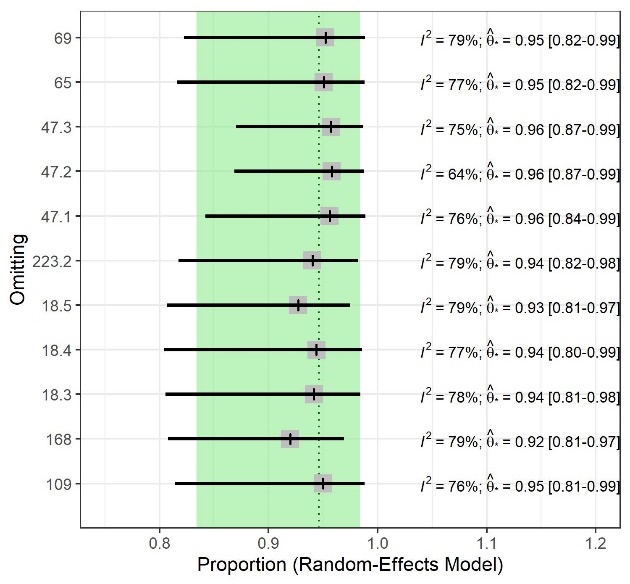

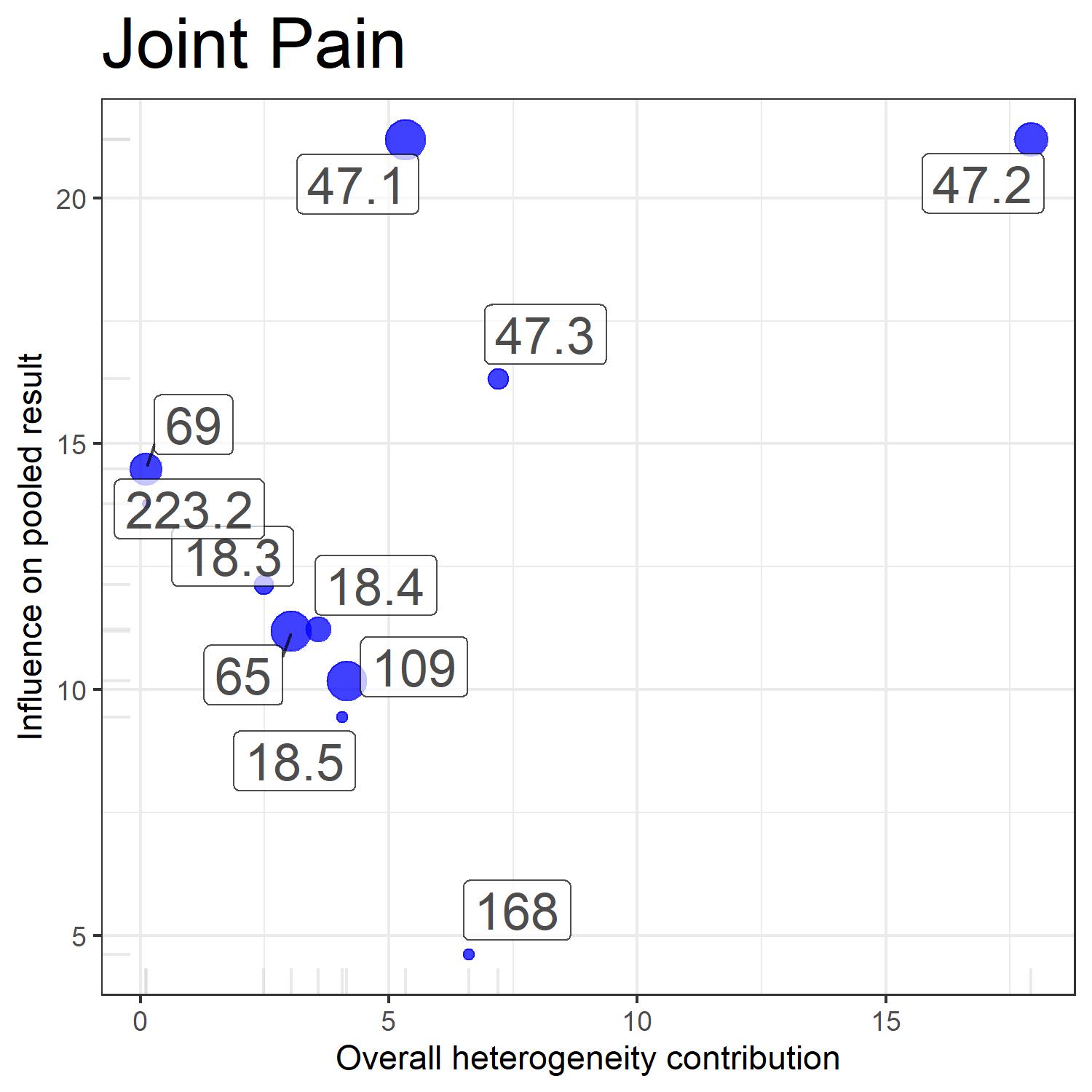


**Joint Swelling**

Figure 8. The analyses to identify outliers and influential groups signalled that the groups reported by Borgherini et al. [48] and Sissoko et al. [168] might be outlying for this endpoint. In the first case, there was no indication that the study would be an outlier. In the latter study, one of the inclusion criteria for the population was fever and/or polyarthralgia, which might suggest that patients who experience polyarthralgia also have more frequent joint swelling. However, the population is deemed to be consistent with the other population, so therefore it was retained in the meta-analysis for joint swelling.


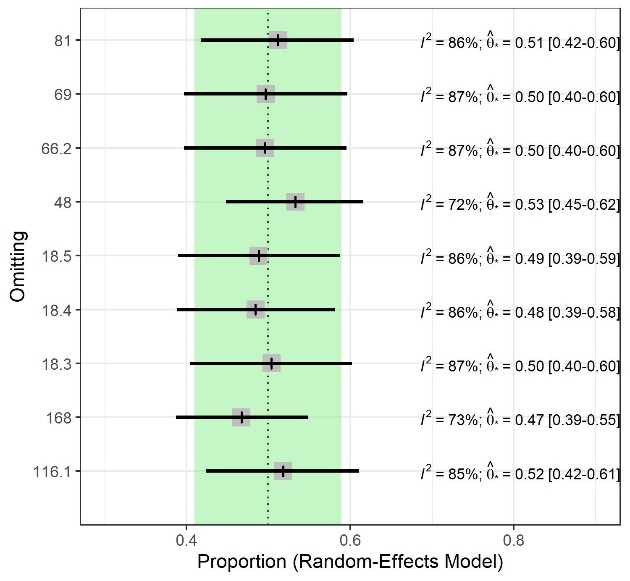

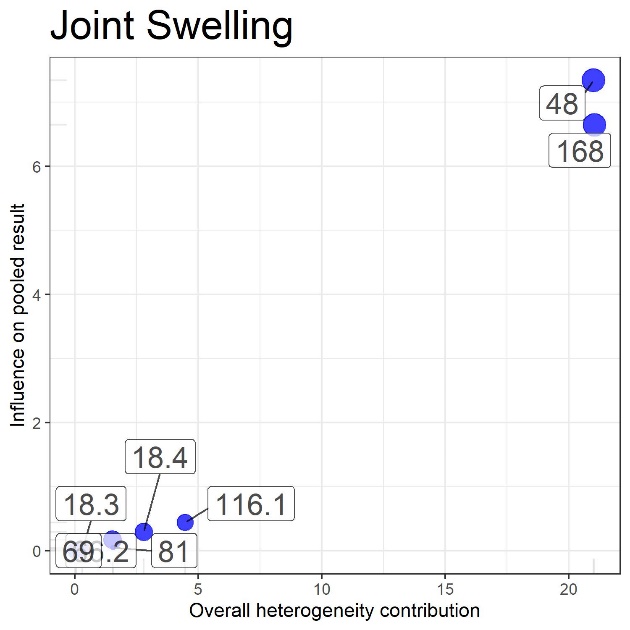


**Myalgia**

Figure 9. In their study, Godeart et al. [211] reported a population that was identified as an outlier for the meta-analysis on myalgia as this group reports a relatively low percentage of patients with this symptom. However, there are no signs in this study that indicate that their population is slightly different from the specified general population. Thus, the study by Godeart et al. was retained for the meta-analysis on myalgia.


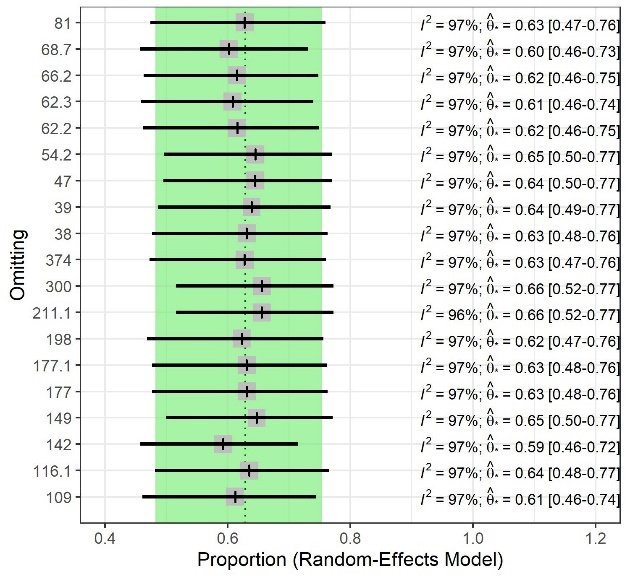

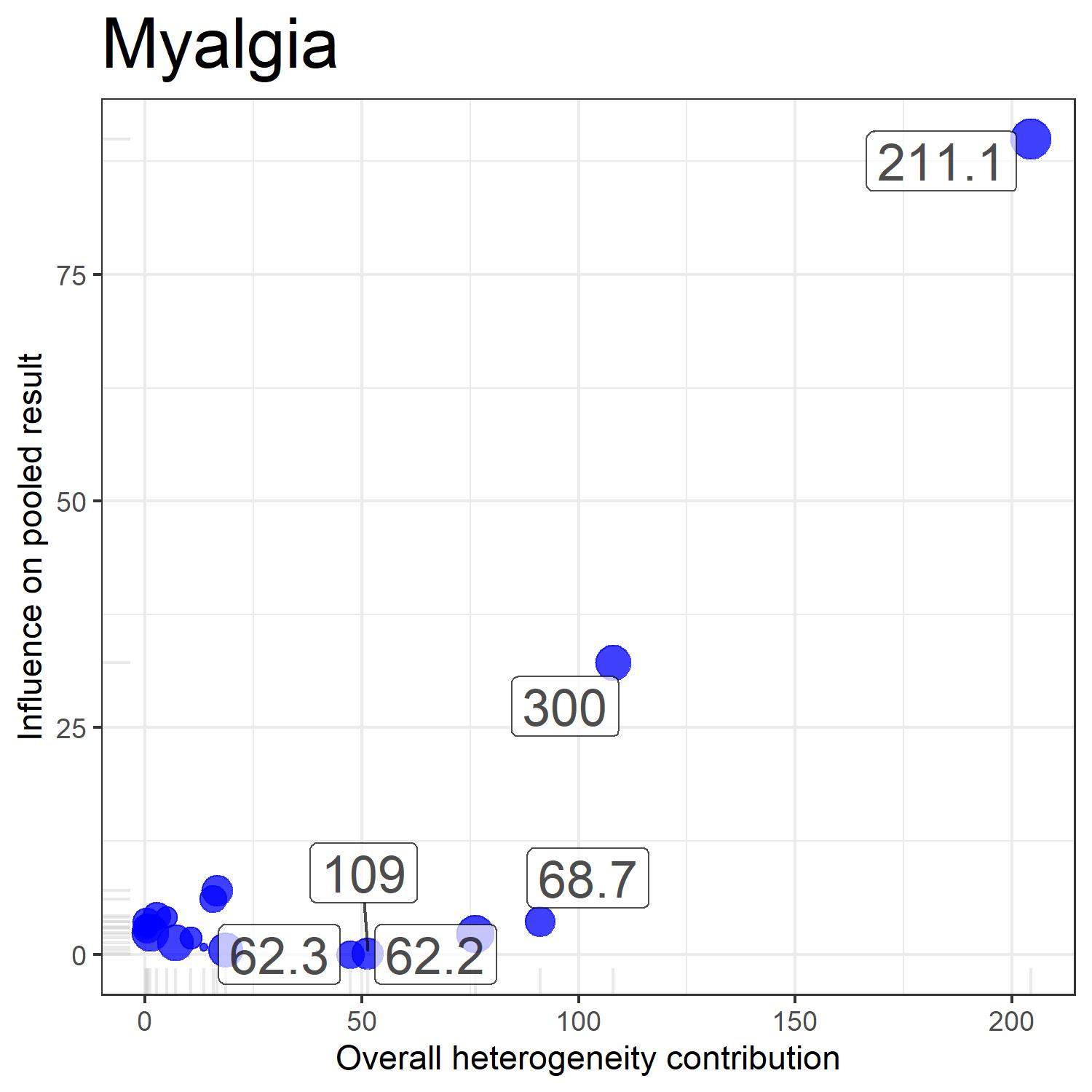


**Nausea**

Figure 10. One study reporting data on two outlying populations was identified in the sensitivity analysis. This study by Chopra et al. [62] was also found to be an outlying study in the meta-analyses on fever and headache. Nevertheless, as mentioned previously, there were no indications that the population analysed in this study differed from the general one. The study was therefore retained in the meta-analysis of nausea.


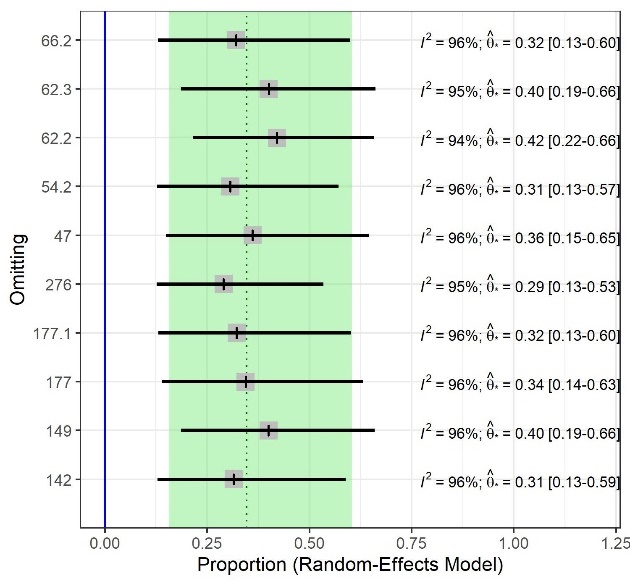

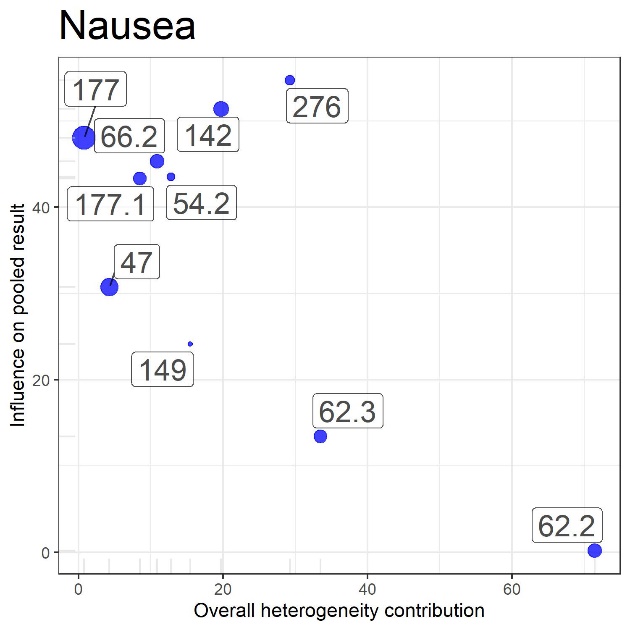


**Rush**

Figure 11. The group reported by Murillo-Zamora et al. [112] was identified as an outlier in the meta-analysis of rash. This study has been identified as an outlier two times before, in the meta-analyses of fatigue and headache. This study aimed to explore the relationship between depressive symptoms and CHIKV infection. However, the included population seemed to be following the description of our general population, so the study was retained for the meta-analysis of rash.


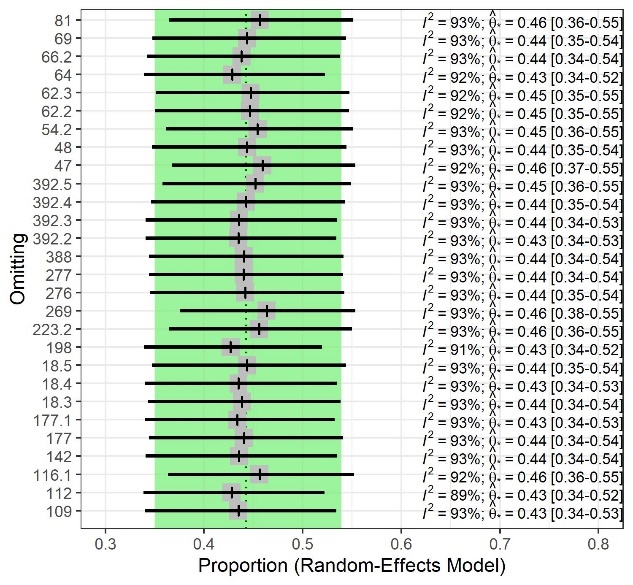

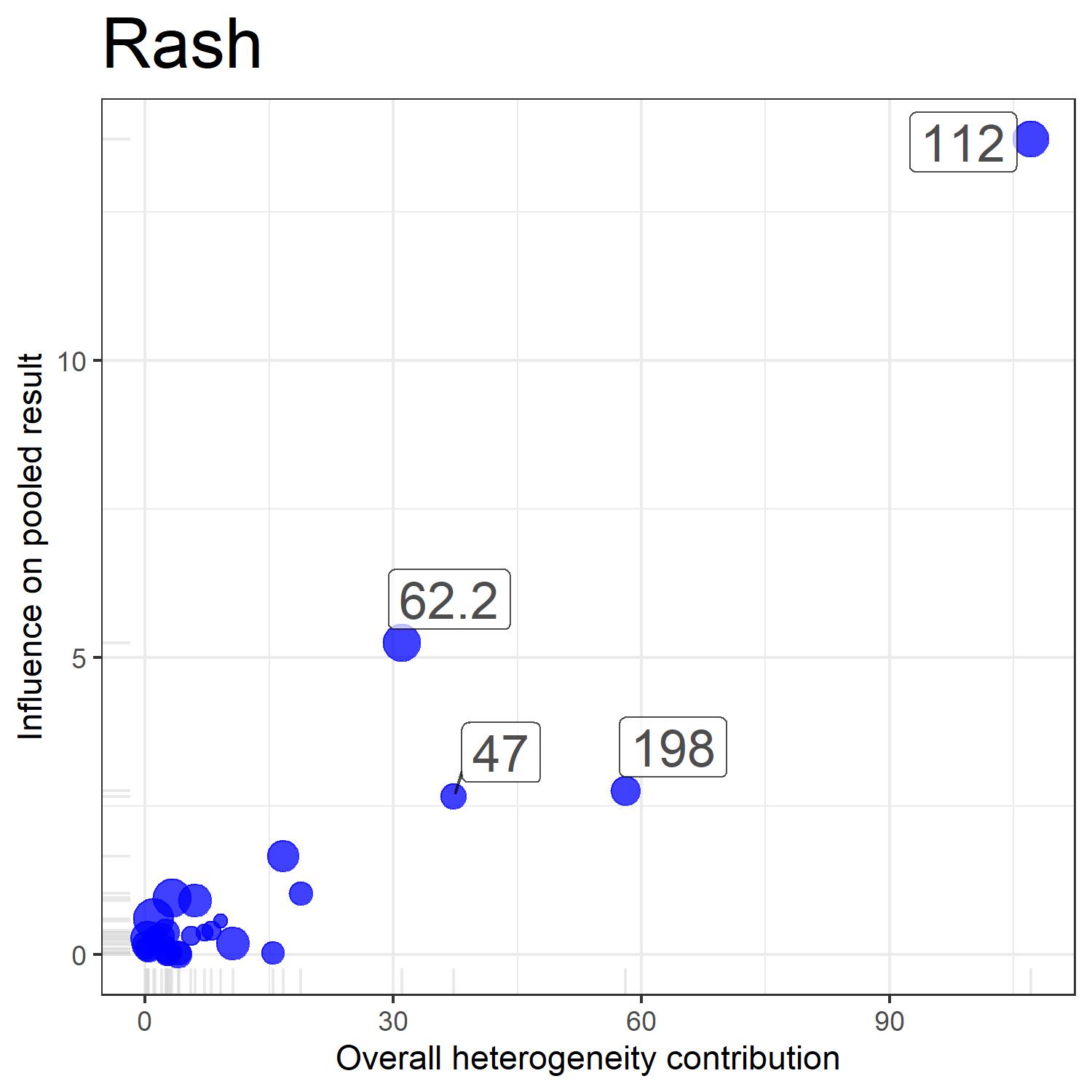


**Vomiting**

Figure 12. Following the sensitivity analysis, three groups were identified as outliers. These were reported by Chopra et al. [62], Imad et al. [66.2], and Jain et al. [69]. However, there is no clear indication in any of these studies as to why they should be selected as outliers. Thus, all studies were retained in the meta-analysis of vomiting.


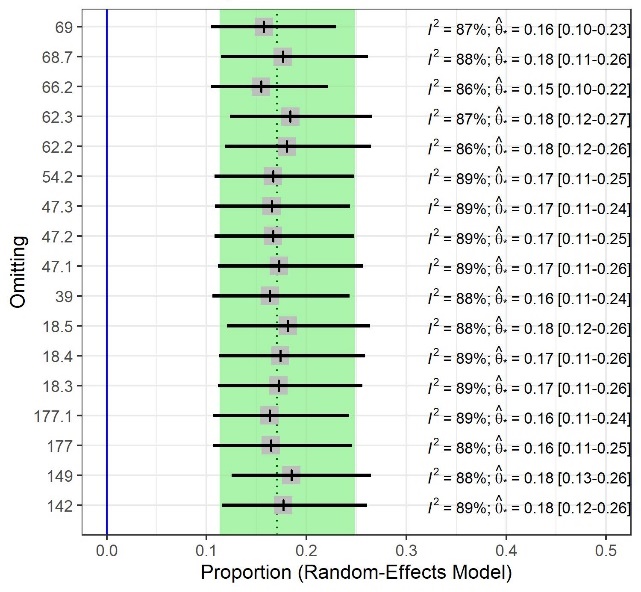

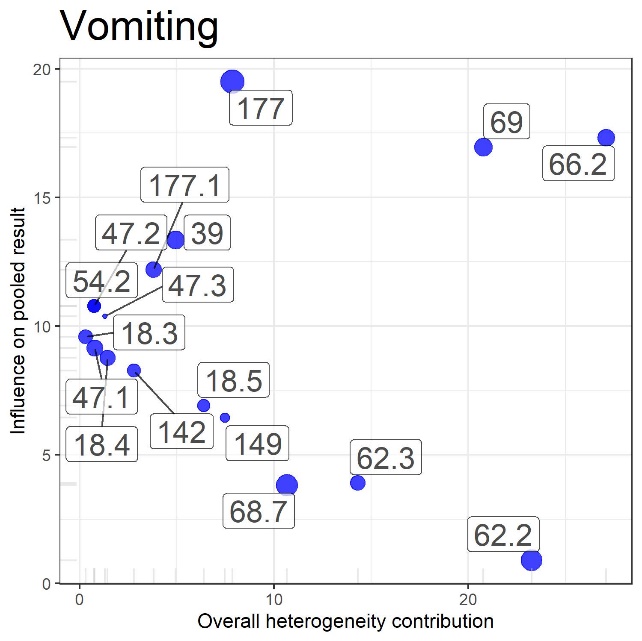


*General Population*

**Mortality Rate**

Figure 13. For the normal-risk subgroup analysis, the studies by Perti et al. [173] from 2016 and Hertz et al. [105] from 2012 were identified as the most influential (Figure 40). Perti et al.'s study involved a population with a mean age of 68.8 years, which may explain the higher reported mortality rate. Similarly, Hertz et al.'s study included inpatients, potentially contributing to the relatively high mortality rate in that study as well. These influential studies were not placed in the higher-risk subgroup because they didn’t follow the specific criteria, however, there are indications as to why their observed mortality rates might be higher than the average


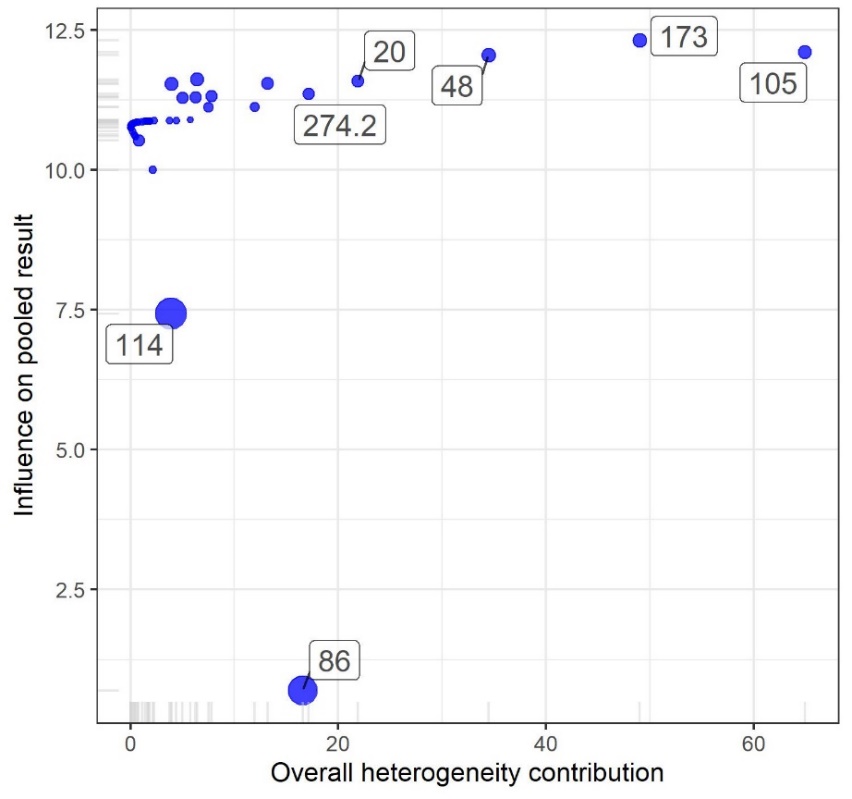

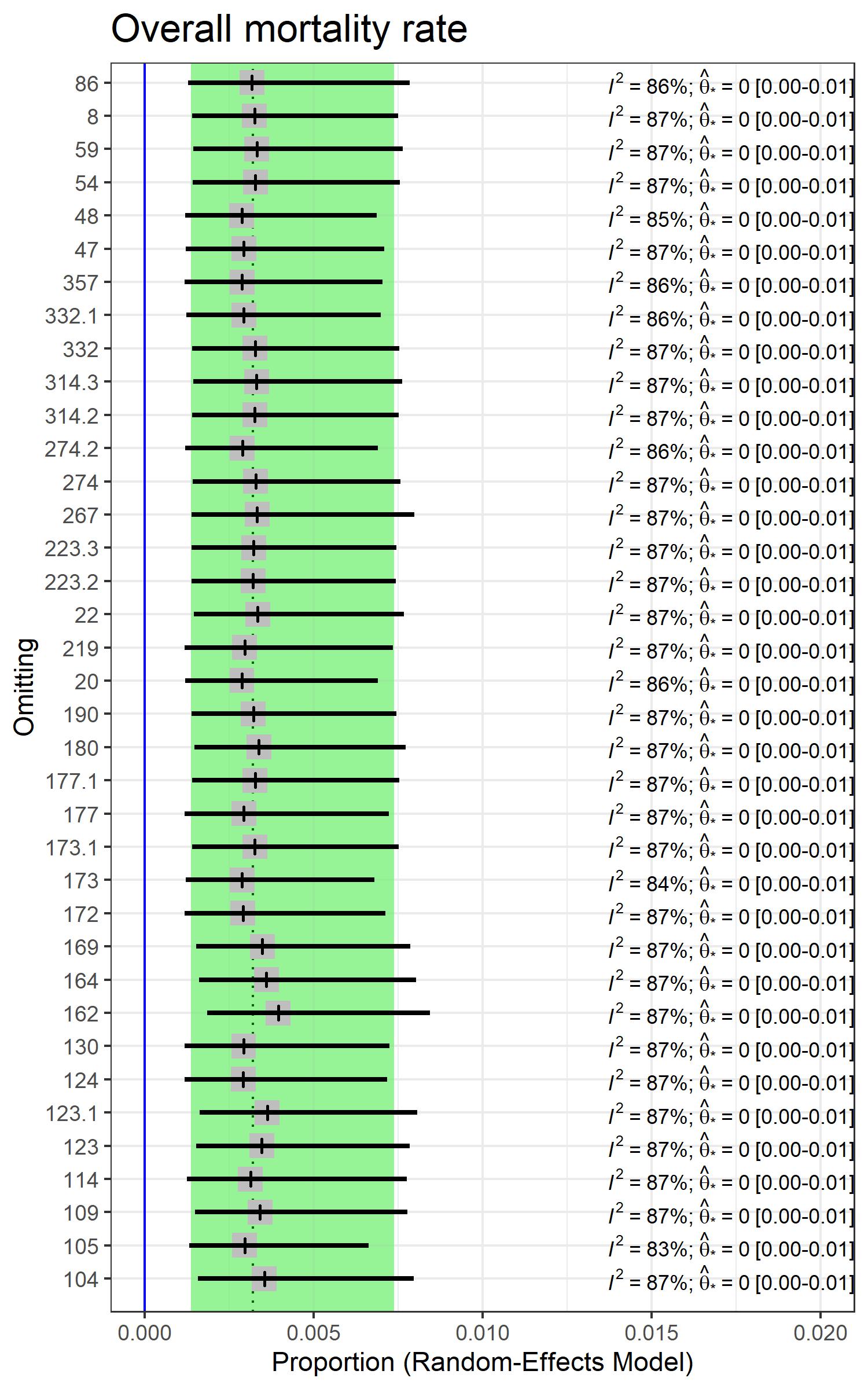


**Mortality Rate (High Risk)**

Figure 14. Both subgroups exhibit heterogeneity, but the high-risk populations demonstrate a higher estimated heterogeneity (I2 = 97%) compared to the normal-risk population (I2 = 87%). Among the high-risk populations, the study by Dorléans et al. [311] from 2018, which included patients admitted to the ED for at least 24 hours, holds the most influence due to its substantial sample size. Consequently, it is considered the most influential study within the high-risk subgroup analysis.


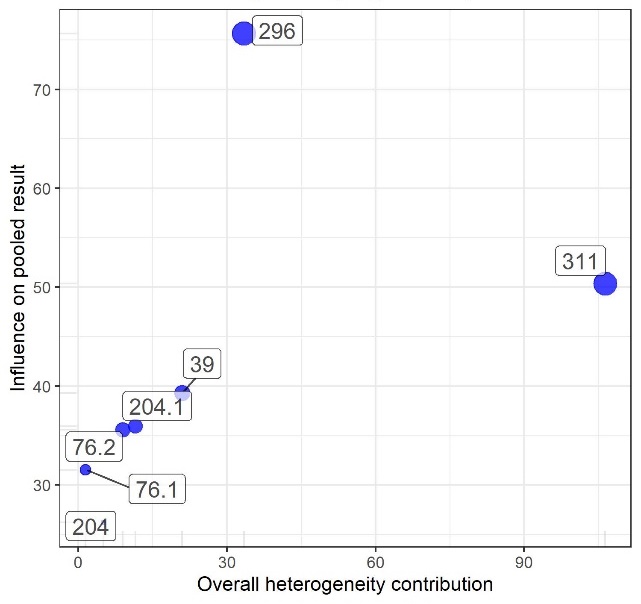

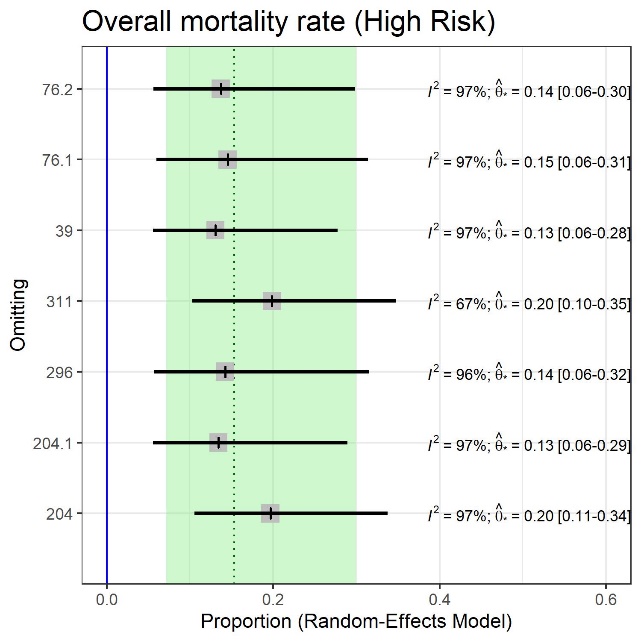


**Chronicity Rate (3 Months)**


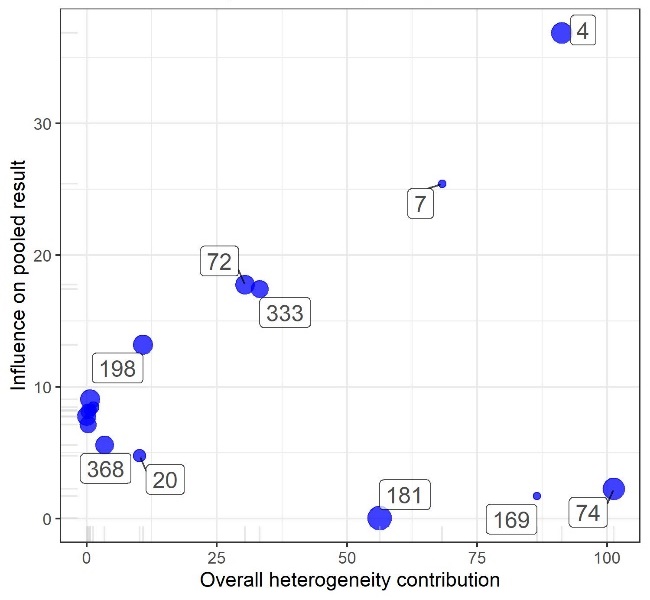

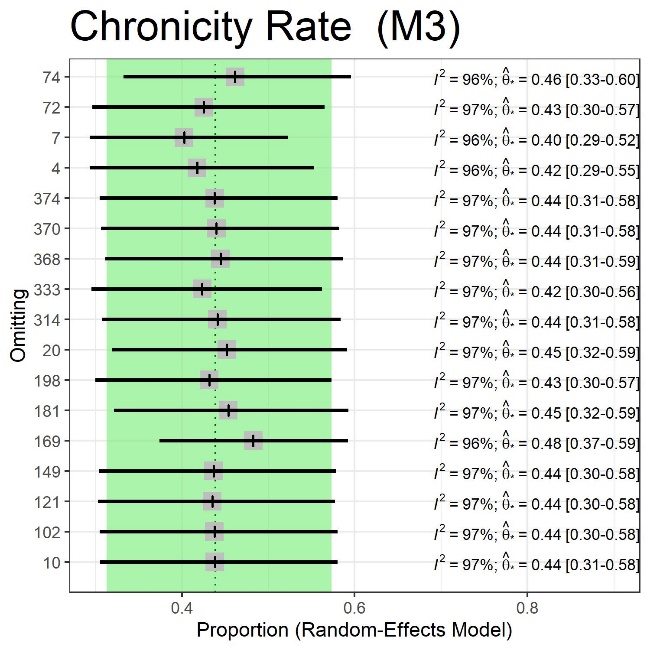


**Chronicity Rate (6 Months)**


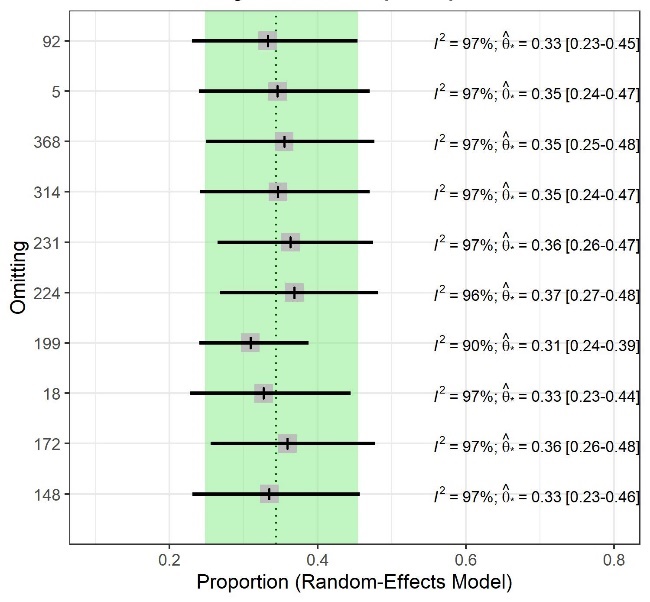

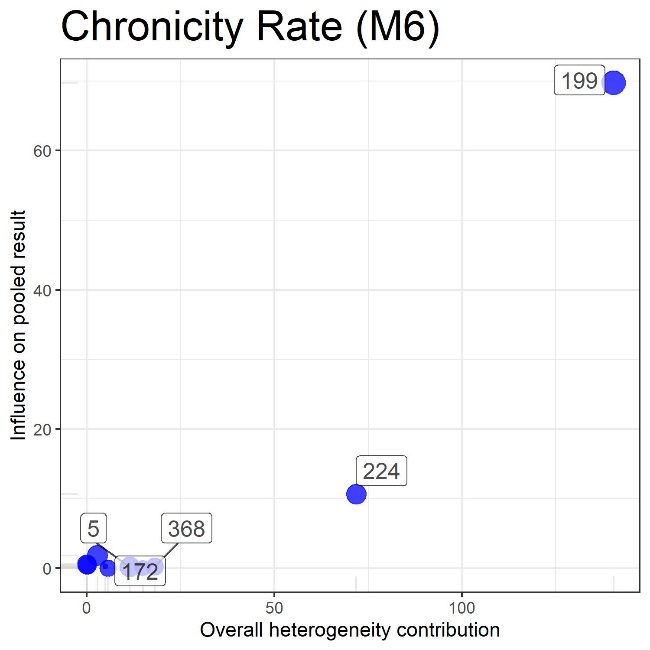


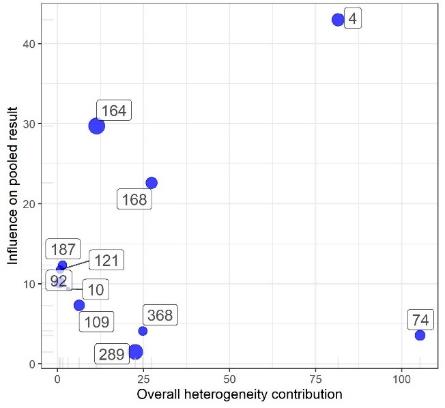

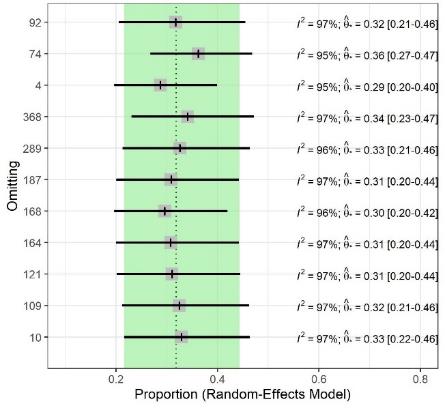
**Chronicity Rate (12 Months)**

Figure 15(a, b, c). In the sensitivity analyses, the studies conducted by Moro et al. [4] and Paul et al. [7] stood out as potential outliers. In Paul et al.'s study, the inclusion involved a patient cohort from an outpatient clinic, but no specific filters were applied to include only chronic patients. Similarly, Moro et al.'s study encompassed a comprehensive representation of the entire population. In both cases, there is not a clear indicator pinpointing the reasons for their outlier status, so both studies were retained within the analyses.

**Symptomatic Rate**

Figure 16. Following the sensitivity analyses, the populations reported by Barreto et al. [218], Centers for Disease Control and Prevention (CDC) [263] and Bustos Carrillo et al. [89] were identified as potential outlier. However, there was no indication that these studies differed from the other populations that were included for this endpoint. Therefore, the studies was retained in the meta-analysis for the symptomatic rate.


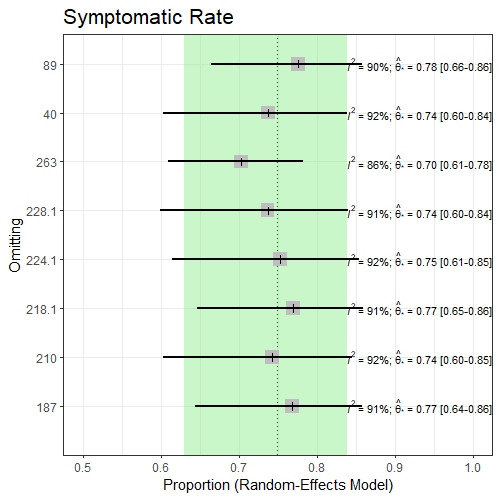

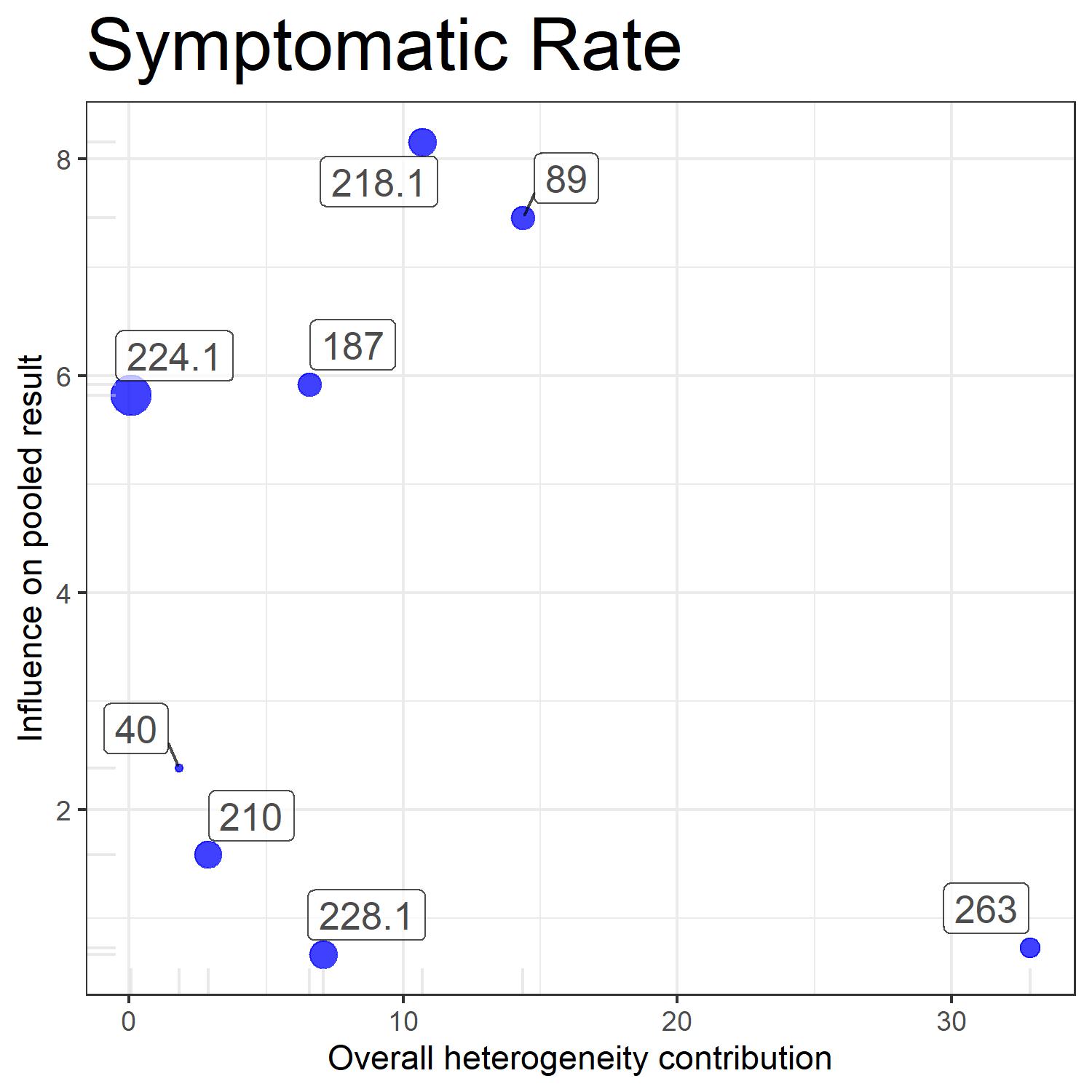

Supplement: S2 Fig — (DOCX) [file pntd.0012254.s007.docx]
