# Supplementary material for: Clinical outcomes of chikungunya: A systematic literature review and meta-analysis
Source: PLoS Negl Trop Dis. 2024 Jun 7;18(6):e0012254. doi: 10.1371/journal.pntd.0012254 (PMC11189168; doi:10.1371/journal.pntd.0012254)
Supplement: Attachment — Submitted filename: reviewers_letter.pdf [file pntd.0012254.s008.pdf]

Response to Reviewers

Kris Rama, Adrianne de Roo, Timon Louwsma
Groningen, The Netherlands

06/05/24

Editor and Reviewers:

We would like to express our gratitude for the time and effort that you have put into reviewing our manuscript titled "Clinical outcomes of chikungunya: a systematic literature review and meta-analysis" and for providing valuable comments. We have carefully considered all the suggestions made by the reviewers and have made revisions to the manuscript accordingly. These changes not only improve the manuscript but also significantly contribute to the important value story we are aiming to convey. We believe that with your feedback, our research now provides a more robust and comprehensive understanding of the clinical outcomes associated with chikungunya.
Please find a detailed response to each comment below, including a description of the changes we have made in the manuscript.

Reviewer 1: Review, Responses & Changes Made

Methods

The objectives were clearly state however a better description of the subpopulations within the text in methods and results would be helpful to add clarity to the study design. Although the results were only based on ∼316 studies, it's not clear whether this number of studies and the sample size within these studies were adequately powered. I recommend that a statistician review the statistical methods in detail.

- **Subpopulations Description**: Thank you for pointing this out. We agreed that the descriptions of the populations were less clear than intended. Therefore, we have updated the description of the subpopulations in both the methods and results section with a more elaborate definition that we hope provides the reader more clarity in our construction of the subpopulations.

- **Sample Size and Power Analysis**: Thank you for the valuable suggestion. We acknowledge the concern about statistical power and we consulted two of our colleagues with a statistical training that reviewed our work, agreed with the methods and deem the results valuable. They reported: "Hoenig and Heisey (2001), among others, highlight that power calculations are best utilized during study design rather than for interpreting results post hoc. Post hoc power analysis has its limitations and does not add value beyond what is gleaned from confidence intervals and p-values" Considering power is more useful during the research design, that is, the choice of the minimum number of studies to include in the each meta-analysis. We included at least five studies to enhance overall precision and reduce the risk of Type II errors. We designed our meta-analysis to maximize statistical power while attempting to provide estimates for most of the endpoints. Yet we remain prudent in drawing conclusions, mindful of the inherent limitations and the possibility of undetected true effects.

Results

The analysis matched the plan however as stated in the methods section, the subpopulations needed to be better described in the text of the manuscript. Page 5 lines 144-147 Please add the percent along with the N for each symptom. For example, rash constituted 171 studies of the 183 studies this would be 93%. Page 5 line 166 The authors report mortality rate as a percentage. Please also calculate the mortality per 100,000 population. Many of the supplemental figures seem key to the authors conclusion yet they are hidden in supplemental figures. My suggestion is the figures most relevant to new findings should be pulled out of supplemental figures and added to the body of the manuscript. For example, adding the plots of those symptoms with the most variability between studies. Figures from the general population in S2 would be worthwhile adding to the main body of the manuscript. Figure 2 indicates a subpopulation of N=756 and then the flow chart bifurcates into exclusion based on newborns and children <15yr. It would be helpful to understand the number of studies excluded in each of these boxes.

- **Subpopulation Details**: We want to thank the reviewer for this valuable feedback. We have clarified the subpopulation details on Page 5, lines 144-147, and included percentages along with N values for each symptom.
- **Mortality Rate Calculation**: We want to thank you for the comment. We have now calculated mortality per 100,000 population as suggested and included this in the results section.
- **Figure Placement**: We really appreciate the suggestion. It was something we considered multiple times in the writing of the manuscript, but decided to place all the forest plots in the appendix. We agree that the main results should be supported by graphs in the main text so we moved

the forest plots of the general population from the supplementary material to the main body to emphasize new findings. We believe this will help the reader more directly follow our story.

Conclusions

The authors aimed to perform a comprehensive meta-analysis for chikungunya specifically focusing on specific symptoms, mortality and chronicity rates observed in both acute and convalescent phases of the illness. The manuscript provides important information however many of the findings are not unexpected. It would be interesting to compare studies with different subtypes (WA, ECSA, IOL, Asia and American subtypes) to determine if the subtypes have differences in symptoms, mortality or chronicity. Also, it would be interesting to compare outbreaks from several decades versus most recent outbreaks to determine if the virus has become more or less pathogenic over the years. Another concern was the extremely high mortality rate in the elderly of 15.4% and 36.67% in specific populations stated in the discussion section. This information does not seem likely considering that chikungunya has not historically caused mortality rates in these ranges even in the largest outbreaks in either Asia, Africa or the Americas. These numbers need to be better explained from where they were derived, the studies indicating these rates and their sample size. Also, whether the death certificates stated CHIKV as the cause of death. Many of the severe cases often are from people who have underlying conditions and the cause of death may not be specifically due to CHIKV. Finally, a better description of the subpopulations within the text in methods and results would be helpful to add clarity to the study design. Although the results were only based on ∼316 studies, its not clear whether this number of studies and the sample size within these studies were adequately powered.

- **Comparative Analysis**: We want to thank the reviewer for their great comments and ideas regarding our study and the disease area. We found both the subtype comparisons and the outbreak comparisons interesting suggestions that highlight very important topics we would like to explore in the future. These study expansions were something we considered during our study, but we were not able to extract enough data for an analysis of this kind while preparing for such a wide meta-analysis, which was our main goal. We believe a new research design is needed to answer those questions, where the researcher would focus on specific endpoints that would highlight the evolution of outbreaks and subtypes. These are very important suggestions for future research, that we will definitely keep in mind.

- **Mortality Rates Explanation**: We appreciate the critical evaluation of the mortality rates reported. We added in the discussion an explanation on how the definition of high risk could have influenced the results. Additionally, we included the forest plot for mortality rate in the main text so that the reader can check the studies used. We acknowledge the difficulty

in differentiating the effects of comorbidities from the direct impact of CHIKV on mortality, especially with inconclusive death certificate data. The possibility of publication bias is also recognized, which might lead to an over-representation of severe CHIKV cases and an overestimation of mortality risk. Despite potential biases and the complexity of attributing cause of death, we indicate that the mortality impact of CHIKV among a high risk group should not be underestimated.

Editorial Suggestions

I suggested that some of the Figures should be placed in the main body of the manuscript instead of only in the supplemental sections.

- **Figures**: Based on this good recommendation, we have moved some figures to the main text.
- **Typos and Grammar**: Thank you for the detailed review. We corrected the typographical errors and grammatical mistakes as pointed out.

Summary and General Comments

See my comments in the conclusion section regarding the describing outbreaks over time and viral subtype analyses.

- **Outbreaks and Viral Subtype Analyses**: We are grateful for this insightful suggestion and its potential to enrich our research. We plan to hold this recommendation in high regard when embarking on subsequent projects. We included a new subsection in the discussion where we suggest that future research could compare different viral subtypes and historical versus recent outbreaks to address the pathogenicity over time.

Reviewer 2: Review, Responses & Changes Made

Methods

No changes were required based on Reviewer 2's feedback for this section.

Results

There needs to be a fuller description of the studies included (apologies if I've missed this in supplementary materials). I appreciate this could become turgid, so care will have to be taken with writing, but readers need to understand a little more about the studies included to critically assess the results. An additional figure might clarify this. I would like to know the distinction between cohort and longitudinal studies, and what intersection there is between the cohort, longitudinal, prospective, and retrospective studies? How and why were

symptoms studied in case-control studies, and what were the purposes of these studies. In addition, further information on specific groups of studies would help. Did the cohort studies (or some of them) follow a non-diseased group until (among some of them) disease developed, and then follow symptoms? I'd also like to know whether any of the studies included a control group.

- **Description of Studies**: Thank you for the valuable feedback. We understand the necessity for a comprehensive description of the studies included to allow readers to critically appraise the results. We would like to clarify that a detailed description can be found in the supplementary materials, specifically in the S2 Table. We acknowledge the concern regarding the potential tedium of an overly exhaustive description within the main text. To strike a balance between informative content and readability, we decided to summarize key aspects while directing readers to the supplementary materials for more detailed information.

- **Case-control, Cohort Study and Control groups**: Thank you for highlighting this. We had four case control studies coming out of our SLR: 42, 65, 165, 377.
  Ballera et al., 2015 [42]: is an outbreak investigation to identify risk factors associated with chikungunya. It reports hospitalization and symptoms for the cases.
  Bonifay et al., 2019 [65]: compares CHIKV-infected patients with DENV patients.
  Sitepu et al., 2020 [165]: same as 42
  Medina-Cintrón et al., 2021 [377]: evaluates the clinical features and correlates in patients with chronic CHIKV arthritis who have not received therapy with DMARDs.
  All of the individuals included in the case-control study had either suspected or confirmed chikungunya virus (CHIKV) infection.
  Control groups were present in two of the experimental studies. We have included a new section in the discussion to clarify their role.

Conclusions

There's an implicit assumption that the symptoms described have a causal association to Chikungunya. Some symptoms such as myalgia and fatigue have quite high population prevalence and may not have a causal association to previously diagnosed CHIKV. I appreciate this can't be overcome in this study, but should be mentioned as a limitation.

- **Symptom Causality**: Thank you for pointing out this important interpretation of the results. We have included the limitations regarding the causal association of symptoms to Chikungunya, particularly myalgia and fatigue now in the discussion.

Editorial Suggestions

At line 114 I think there is a typographical error; "commodities" should be "co-morbidities". At line 141, substitute "one" for "less". It's better grammatically and more precise (assuming there weren't zero papers from some of these areas). At line 264, what does "...challenges due to lack and/or data quality" mean? It needs to be rewritten. I wonder if the meaning is "poor quality or absent data"?

- **Clarifications and Corrections**: Thank you for your observations. The term "commodities" is now correctly stated as "comorbidities," the term "one paper" has been used for accuracy, and the passage has been clarified to "challenges due to poor or absent data quality."

Summary and General Comments

Please discuss critically your statement regarding the prevalence of arthralgia and arthritis (lines 181-2), including the definition for the latter. The definition of arthritis that I'm familiar with requires physician examination. Unless all studies included examination by a clinician this would be an important bias in estimation of arthritis prevalence. If none of the studies involved physician examination then arthritis could not be determined. You state that you found 11 experimental studies (line 113). More information is needed on these. Were they the 11 studies on co-infection with Zika and Chikungunya? What was the study design? Were they clinical or epidemiological studies? If the latter, presumably randomised control trials?

- **Arthritis definition**: Thank you for prompting a critical evaluation arthritis definition. However, our analysis was constrained by the definitions provided in the original studies. These studies did not uniformly include physician examinations as part of their diagnostic criteria for arthritis, which we acknowledged might affect the reported prevalence rates. Given the reliance on the original studies' definitions and the absence of consistent physical verification by a physician, we used the data as presented.
- **Experimental studies**: We appreciate the insightful comments and suggestions for additional details. These experimental studies were not focused on co-infection with Zika and Chikungunya. However, we recognize that the study designs and their are not clearly stated, and we have added more information on the experimental studies in the text. Additionally, we provide a new sheet in S2 Table highlights the details about the experimental studies. We hope this will allow readers to quickly assess the experimental studies if interested.

Reviewer 3: Review, Responses & Changes Made

Methods

S2 Checklist is incomplete. Please provide complete PRISMA checklist. No other concerns about the Methods. The inclusion criteria are very broad, but this is acknowledged by the authors and probably necessary to cover the full range of symptomatology and clinical outcomes.

- **PRISMA Checklist**: Thank you for the careful review. We have completed the PRISMA checklist as requested and included it in the revised supplementary material.

Results

Results are well presented in figures and tables. No further recommendations.

No changes were required based on Reviewer 3's feedback for this section.

Conclusions

Conclusions are not groundbreaking but further affirm most prevalent and frequent symptoms during CHIKV infection, and also provide additional data on chronicity of CHIKV infection and hospitalisation/ mortality rates.

No changes were required based on Reviewer 3's feedback for this section.

Editorial Suggestions

None - just to update table S2 and/ or include completed PRISMA diagram as best practice.

- **PRISMA Diagram**: Updated Table S2 and included the completed PRISMA diagram.

Summary and General Comments

Well-written study that provides additional evidence on the clinical endpoints of CHIKV. Line 310: 's' missing on 'endpoint'.

- **Grammar Correction**: Thank you for pointing this out. We fixed the missing 's' on 'endpoint' as indicated.

In summary, we believe that the revisions made have addressed the concerns raised by the reviewers, and we hope that the manuscript is now suitable for publication. We have also ensured that all data underlying the findings are fully available without restriction and that all figures meet PLOS requirements.

Thank you once again for your constructive comments, which have undoubtedly improved the quality of the manuscript.

Sincerely,

Kris Rama, Adrianne de Roo, Timon Louwsma
